# Cross-layer latency analysis for 5G NR in V2X communications

**DOI:** 10.1371/journal.pone.0313772

**Published:** 2025-01-09

**Authors:** Jorge Horta, Mario Siller, Salvador Villarreal-Reyes

**Affiliations:** 1 CINVESTAV Unidad Guadalajara, Zapopan, Jalisco, Mexico; 2 CICESE, Ensenada, Baja California, Mexico; University of Vaasa: Vaasan Yliopisto, FINLAND

## Abstract

The 5G network was developed to push the capabilities of wireless networks to previously unseen performance limits, e.g., transmission rates of several gigabits per second, latency of less than a millisecond, and millions of devices connected at the same time. To meet these requirements, it is necessary to access new spectrum (the so-called millimeter waves) and use techniques such as Massive MIMO (Multiple-Input Multiple-Output) and beamforming. This required the design of a new radio interface, known as 5G NR, that includes improvements to its physical components and new protocols. The performance of the 5G network will depend heavily on the behavior of these new protocols under certain configuration parameters, traffic conditions, device density, and network architecture. This paper introduces an analytical model for the performance evaluation of 5G NR. The developed model describes the behavior of the different layer 1 and 2 protocols involved in 5G radio communication. Using the model, it is possible to evaluate the performance of 5G NR in terms of throughput and latency, two key performance metrics used to describe QoS (*Quality of Service*) thresholds of different applications. The protocol layer approach gives the model sufficient granularity to identify critical behaviors that significantly impact performance. This can help focus efforts on improving these key points or propose improvements/modifications to the operation of network protocols or devices. The use of this model for performance evaluation is exemplified by studying a Remote Driving scenario operated over 5G. This scenario has very stringent delay requirements, which, according to the model’s results, can be satisfied if the network conditions are adequate. This model and its results can be used as a starting point for performance evaluations of application involving end-to-end (E2E) communications.

## 1 Introduction

**Fig 1:**
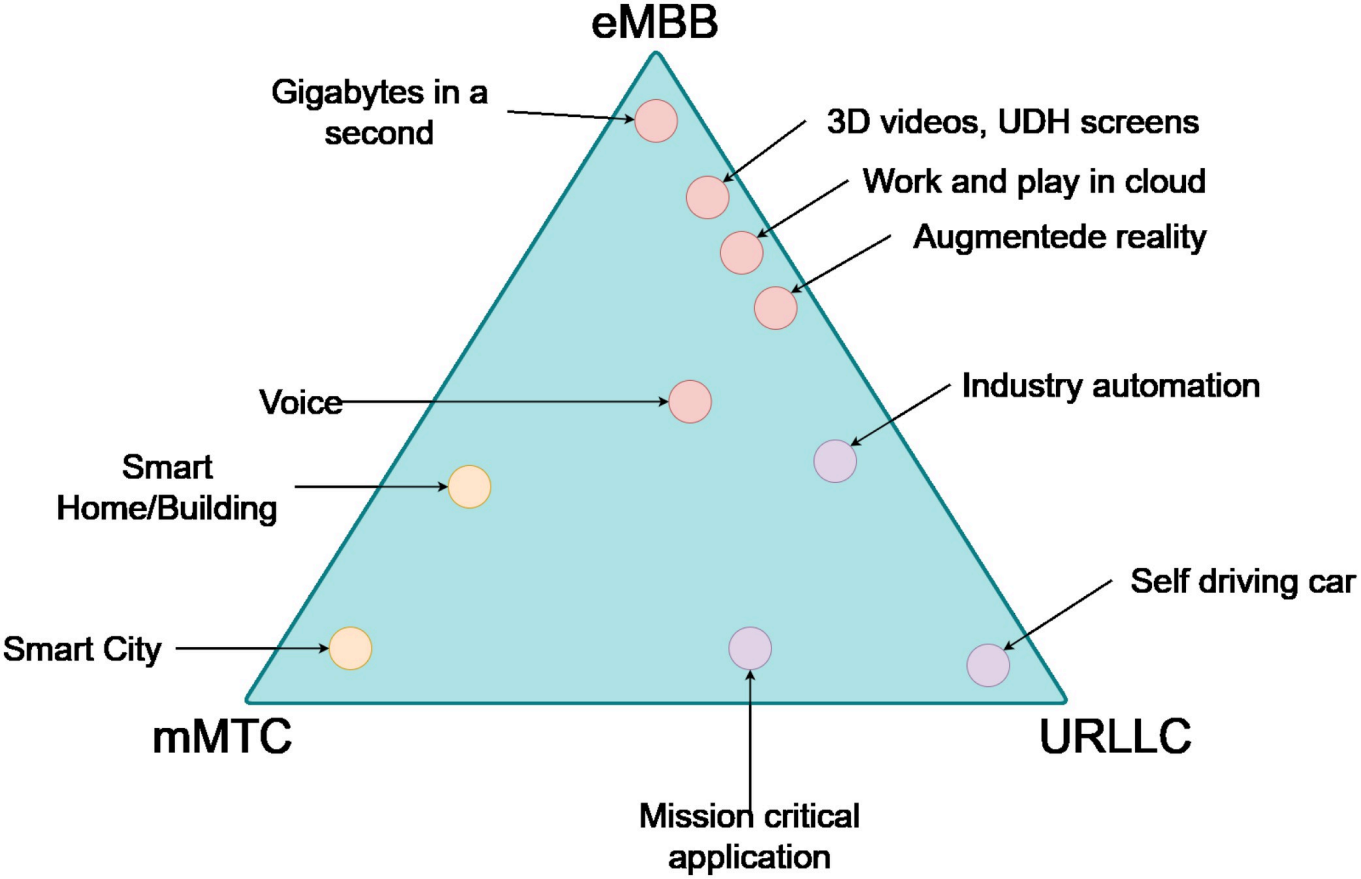
IMT-2020 use case and scenarios [4].

The fifth-generation (5G) of mobile networks aim to provide performance that significantly exceeds the one offered by fourth-generation (4G) networks. For instance, provisioning of peak data rates of 20 Gbps, latencies of 1ms, successful transmission probabilities above 99.999%, high density of connected devices (up to 1000000 devices/*km*^2^), and improved network performance (Table 1.1 in [1]), should be feasible with 5G deployments. Different organizations used these new performance limits to define use cases to guide the development of 5G technology. For instance, IMT-2020 defines three key use cases available for 5G. These cases, shown in Fig 1, are Enhanced Mobile Bandwidth (eMBB), Massive Machine Type Communications (mMTC), and Ultra Reliable Low Latency Communications (URLLC).

The IMT-2020 classification represents these use cases as a triangle, with each vertex representing a case with different requirements. The eMBB case focuses on improving the transmission rate, mMTC increasing the number of connected devices, and URLLC reducing latency. Different applications can be placed in this triangle depending on the combination of their specific requirements. Consider the mission-critical and self-driving car applications, both of which require a low delay time, thus they are located near the URLLC vertex. The Smart City application favors connecting more devices simultaneously, hence it is located close to the URLLC vertex. On the other hand, video applications, remote work, or augmented reality require higher bandwidth. To achieve these performance goals, 5G standardization bodies have defined a New Radio (*NR*) interface which comprises access to new spectrum, massive multi-input multi-output beamforming, network slicing, dual connectivity with 4G, and cloud and edge computing support [2]. Thus, compared to 4G, the introduction of 5G NR required the development of a new protocol stack (layers 1 and 2) to integrate and access these technologies. In addition, in 5G, there is the possibility of enabling edge computing deployments, which brings the processing closer to the user and allows the most demanding performance limits (such as URLLC [3]) to be met. Although protocols and deployment scenarios are designed to ensure that applications meet specific Quality of Service (QoS) metrics, varying network conditions may make this unsatisfactory. It is necessary to evaluate the network configuration under different conditions to foresee behavior and make adjustments to solve it. Analytical models are attractive tools for this.

This paper presents an analytical model for performance evaluation of the 5G NR protocol stack. This model is developed using a layered approach to consider the individual behavior of all protocols in the stack associated with 5G NR communication. The model focuses on describing in sufficient detail the contribution each of the stack’s protocols has on performance. The developed model is used to evaluate the performance of an application deployed in 5G with demanding delay requirements: Remote Driving. The main objectives of this work are:

- Identify the behavior of 5G NR layer 1 and 2 protocols.
- Develop analytical models for each of these protocols.
- Integrate the models into a 5G NR model that considers all the individual contributions of the protocols.
- Conduct a performance evaluation for the remote driving application deployed in 5G.

The rest of the paper is organized as follows: Section 2 briefly introduces the architecture of a network for Remote Driving applications supported by 5G, and the state-of-the-art works relevant to it. Section 3 presents the development of the model, with a focus on analyzing and modeling the different protocols involved in radio communication. Section 4 reports the performance evaluation results for a remote driving application deployed in 5G NR. Finally, Section 6 discusses the developed model and the results obtained using it and concludes the paper.

## 2 Background and related work

Remote driving, part of teleoperation systems, allows a driver (either human or an app) to control a vehicle remotely. The 5G Automotive Association (*5GAA*) describes teleoperated driving as a use case associated with the autonomous driving group. Specifically, teleoperated driving is a Cellular V2X (*C-V2X*) use case in which a remote driver takes control of a vehicle to drive it efficiently and safely from the current location to its destination [5]. Teleoperated systems are mainly made up of three elements: a robot with sensors and actuators that allow the operator to assess the environment and perform actions, generally with one or more cameras; a communication element, usually a wireless network, that allows the robot and the operator to exchange sensor data and control commands; and a control station, which enables the operator to view and interpret sensing and video data as well as input devices that allow the operator to send control commands [6]. Translating this to the 5G remote driving use case, it follows that:

- Robot: It is a system located within the vehicle that is capable of interfacing with control components (i.e., steering wheel, brake, throttle, etc.), in addition to one or more cameras and sensors that allow it to send information about the vehicle state and its surroundings.
- Communication element: The 5G network establishes a communication link between the robot and the operator.
- Control station: The remote site from which the operator will control the vehicle. It is usually equipped with one or several display elements to show the operator the video and data sensed by the vehicle and input devices that will enable commands to be captured and sent (i.e., keyboard, joystick, racing wheel, etc.).

**Fig 2:**
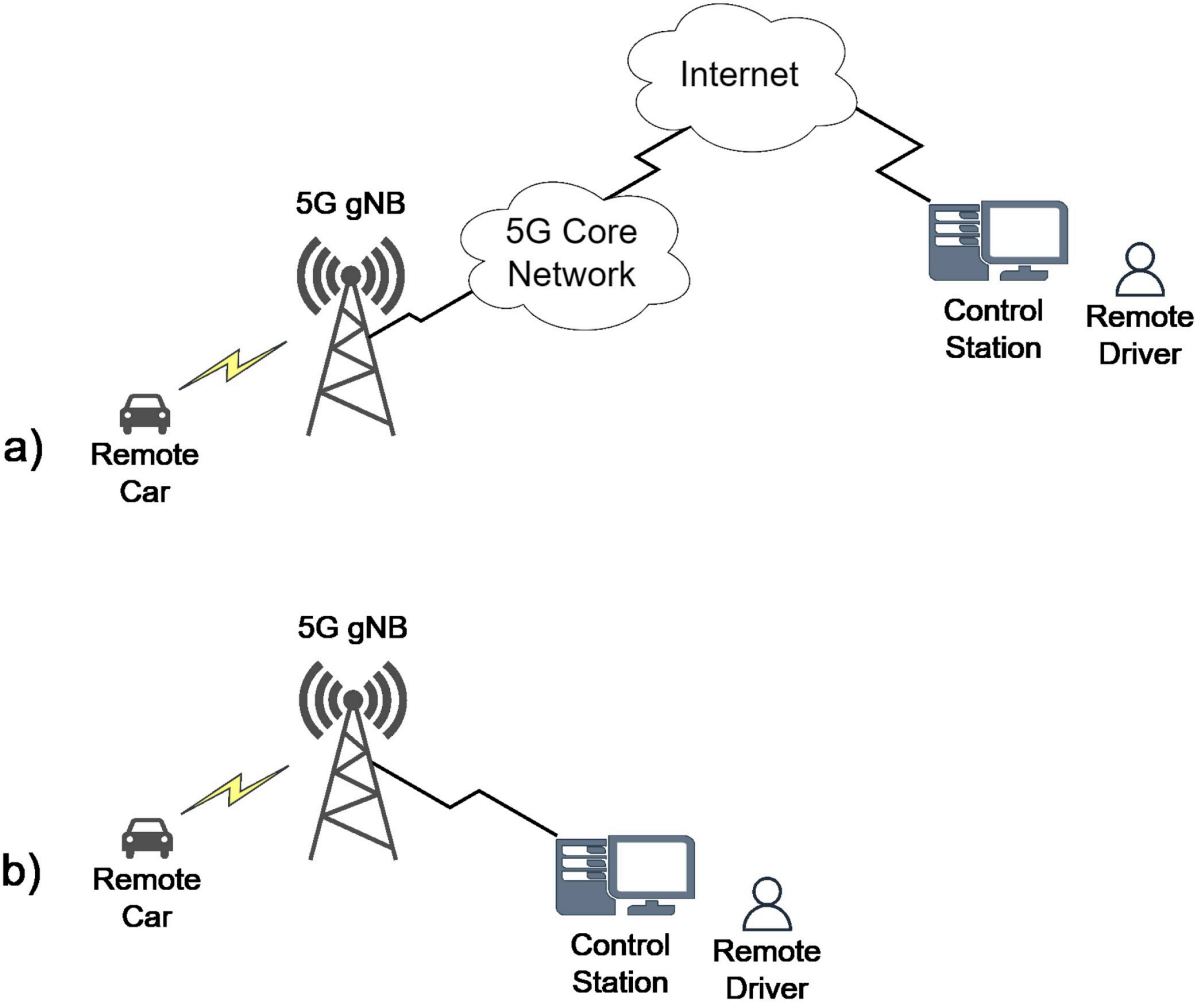
Architecture for a 5G-enabled centralized remote driving application.

The architecture for a centralized remote driving application built with the above elements is shown in Fig 2a. Centralized deployment adds delays associated with traversing the 5G core network and the Internet. These delays may compromise fulfilling the stringent requirements of the remote driving application. Thus, the 5GAA proposed deployment options based on edge computing [7]. They aim to bring computing close to the vehicles to reduce the network delay. Based on these deployments, the control station for remote driving can be located on a node in the 5G Core Network or a site adjacent to the 5G gNB (Fig 2b). This can be considered as the baseline scenario for delay evaluation, because if delay requirements are not fulfilled for this case they will not be fulfilled for deployments where the control station is located further away from the 5G gNB. The evaluation requires estimating the 5G NR link delay under different network conditions and configurations. Then, based on the obtained results, it is possible to decide on the location of the control station that meets the delay requirements for remote driving. Thus, to demonstrate its usefulness, the analytical model introduced in this work is used to evaluate the delay for a remote driving application deployed in a Multi-access Edge Computing (*MEC*) 5G NR deployment where the control station is located next to the gNB (see Fig 2b). However, it is important to note that the use of the model is not limited to remote driving applications.

Several state-of-the-art articles have investigated the application of remote driving, as described below. The authors in [8] present a study of a remote driving application performance and driving experience through an LTE network. The authors develop a prototype of a system to simulate remote driving under different network delay conditions. This prototype driving task is based on video streams transmitted from the prototype vehicle to the remote driving station. LTE network delay conditions are emulated using a probability distribution obtained from field measurements. The objective is to compare the performance and the driving experience for two different scenarios: random delay and constant delay. In the first case, video frames are displayed as soon as they arrive. Thus, they may experience jitter. In the second case, the video frames are delayed to match the maximum delay experienced by the network (358 ms). This is done to eliminate jitter and smooth out the displayed video. The results show that while network delay is the biggest challenge for remote driving, a scenario with high variability (*jitter*) negatively impacts driver performance. They found that the performance in a scenario with constant high delay (with no jitter) is similar to that observed in a scenario with no delay. Conversely, a variable delay scenario imposes a more significant mental and physical load, frustration, and effort on the driver. This is why it is concluded that reducing the network delay could be helpful, but achieving a stable network delay value might be a preferable enhancement for remote driving.

In [9], the authors design and evaluate a remote driving system supported by 4G and 5G networks. This work describes an architecture that allows the application of remote driving and implement this architecture in a field test using a Hardware-In-the-Loop (HiL) simulation. Both implementations are used to evaluate remote driving application delay, bandwidth, and reliability using 4G and 5G networks. The following use cases are considered: straight-line driving and slalom. Those cases are evaluated under different latency and packet loss probability conditions. For remote driving, the vehicle must transmit video and some control commands. This data is presented to the remote driver to make decisions and execute actions sent to the vehicle as control commands. After analyzing the results obtained, the authors concluded that 5G offers advantages over 4G in remote driving applications,i.e. 5G latency is half of 4G latency. Furthermore, they did not observe a strong correlation between network delay and driver performance. They conclude that remote driving applications can be feasible with current technology in a low-speed (less than 40 km/h) scenarios.

In [10], the authors propose a framework for driving vehicles remotely and validate this framework through performance evaluation in a real network environment. To implement this framework, they propose an architecture in which the vehicle is connected to the remote driving station via a commercial 4G/5G network. The architecture includes a remote driving station and a vehicle capable of transmitting video and other signals. It also requires a control mechanism capable of implementing the control commands received from the driver into the vehicle. These requirements are met using an Openpilot system in the test vehicle (2019 Toyota Prius Hybrid) as a basis. The authors modified the Openpilot base system to provide the vehicle with all the functions required to be driven by a remote driver. Using this implementation, two different scenarios are tested: a local and a remote scenario. In the first scenario, the driver and the vehicle are connected to the same wireless network, which offers the best case in terms of latency. In the second scenario, driving uses the commercially available 4G/5G network. After carrying out the different tests and analyzing the results, they conclude that control commands (sent from the remote driver to the vehicle) can experience an average delay of up to 32 ms and still allow remote driving in real-time. On the other hand, the average delay for streaming video is 680 ms, which would increase the difficulty of driving the vehicle remotely. In both cases, network performance is the main cause of delay, so the authors conclude that these results can be improved with the next generation of mobile networks.

The work [11] carries out a study on the remote control of a vehicle using video streams through wireless networks. Using a vehicle model and implementing a system based on the Robot Operating System (*ROS*), remote driving is enabled through a WiFi network that connects the vehicle and the remote driver. Driving tasks are guided by video streamed by the vehicle. The tests use three video-transmission protocols: ROS multi-computer communication, UDP, and TCP. Additionally, an experiment is carried out in which the vehicle is driven based on the direct observation of the driver, which eliminates video delay and allows the impact of network latency to be measured. The different video transmission protocols are evaluated in scenarios with different vehicle speeds. From the results, the authors conclude that remote vehicle operation is feasible if a low vehicle speed is maintained. It was also identified that the UDP-based stream offers the lowest latency for high-resolution video transmission compared to ROS and TCP. Another result found is that driver performance is more affected by delay jumps; thus, achieving a “deterministic delay” with low or no jitter is more important than a low delay with jitter.

Much of the work related to remote driving is oriented towards experimental evaluation of driver performance, and aims to test the feasibility of such applications with current technologies. Regarding analytical modeling, looking at works addressing Vehicle-to-Everything (*VX2*) scenarios is necessary. For instance, [12] presents an analytical 5G NR latency model in a V2X scenario where Vehicle-to-Network-to-Vehicle (*V2N2V*) communication is implemented. This model evaluates latency only at the radio level. The model considers different numerologies (sub-carrier spacing or *SCS*, slot, and symbol duration, and Cyclic Prefixes), modulation and coding schemes, use of slots or mini-slots, dynamic or semi-static scheduling, different re-transmission mechanisms, as well as unicast or broadcast/multicast transmissions under different traffic conditions. The authors use the model to assess the impact of different configurations on 5G delay and identify which ones meet the stringent latency and reliability requirements of V2X. This study is based on a cooperative lane change scenario enabled by V2N2V communication. The evaluation is carried out considering the requirements established by 3GPP associated with Low Level of Automation (*LLoA*) and High Level of Automation (*HLoA*) [13]. These requirements are latencies of 25ms with 90% reliability for LLoA and 6ms with 99.99% reliability. From their results, the authors conclude that, at least at the radio level, 5G can be used for V2X services in an LLoA and periodic traffic environment. This is because all the evaluated scenarios had a latency of less than 6 ms in 90% of the cases. To comply with HLoA requirements, HARQ retransmissions are used. It is necessary to select the appropriate parameters (i.e., SCS and mini-slot) so as not to increase the radio latency and the required bandwidth. The impact of scheduling mechanisms on performance was also investigated. It was identified that semi-static scheduling is adequate to transmit periodic messages, while dynamic programming is more spectrum efficient for aperiodic messages. In the latter case, they demonstrated that the required control command exchange significantly affects the delay. Finally, the authors conclude that V2N2V 5G communication is suitable for V2X applications with aperiodic traffic and non-strict latency requirements if the network load is low or medium and a high SCS value is used.

The work [14] presents a model for V2X application delay in 5G. As an extension of [12], different 5G deployment scenarios are considered to enable V2X applications. The authors argue that the delay is affected by the configuration of the 5G network, the traffic load, and the deployment and location of the application server (*AS*) that hosts the V2X application. In this case, the flexibility of 5G allows different deployment scenarios where the AS can be located in a remote cloud (centralized), or it may be found somewhere in the 5G core or transport networks and even be co-located with the gNB. Deployment scenarios for a cooperative lane change case are evaluated using the traffic and configurations introduced in [12]. Based on the evaluation results, the authors concluded that a centralized scenario (cloud AS) has difficulties meeting the strict delay and reliability requirements of V2X. It was also identified that locating the AS closer to the edge of the cell can reduce delay. Still, the configuration must be chosen carefully, and network dimensioning must be considered. Furthermore, [12] does not address the development of analytical models to calculate the delay introduced by the protocol stack in 5G NR. This paper addresses this issue and thus complements the results presented in [12].

Regarding the handling of different QoS profiles in 5G service we have some works, such as [36]. This paper proposes some Configured Grant (CG) scheduling algorithms that can be adapted to the strict requirements of URLLC. The proposed algorithms, sorted-OFDMA and Best-MatchOFDMA, RB utilization are evaluated under different conditions of packet size, numerology and allocated bandwidth. These algorithms are compared against the traditional 5G algorithm for CG, called SymOFDMA. The results presented seem to indicate that the proposed algorithms have a similar level of efficiency to SymOFDMA.

The work [37] studies the QoS requirements for remote and automated driving in 5G. The author proposes prediction algorithms for adjust the QoS requirements to varying network conditions. These variant conditions depend on background traffic (non-driving related applications) from connected vehicles in the same cell and adjacent cells. This should be reflected in the MAC layer scheduling mechanisms, where traffic can be differentiated. Using the Random Forest algorithm, predictions are generated for different conditions (number of vehicle, positions and network loads) and different prediction windows. By simulation the scenario, it is concluded that the prediction algorithm performs adequately as long as the prediction window is a few seconds and degrades as the window grows.

The authors of [38] propose a parametric model for evaluating the performance of teleoperated driving. Three application scenarios with different requirements are proposed: Driving, Parking and Supervision. An analytical model for QoE based on different KPIs for the main aspects influencing remote driving is developed. These are: Video Coding Quality, Macroblocking and Delay. Using data sets obtained from 4G and 5G networks measured in the corridor between Spain and Portugal, the different KPIs are estimated for different network configurations and conditions. Based on the results obtained, the authors conclude that current networks could hardly meet the requirements of teleoperated driving. However, 5G Stand Alone deployments, dedicated channels (network slicing), and MIMO provided by new chipsets could help meet these requirements.

The scheduling and correct use of radio resources are critical to meeting the requirements of 5G applications and use cases. Therefore, the problem of scheduling and resource allocation has been addressed by different works in the state of the art. The work [39] proposes a resource allocation method aimed at jointly optimizing delay and power consumption in LTE-A networks. This allocation uses the DELFBDO (delay and energyaware Levy flight Brownian movement-based dragonfly optimization) algorithm to define a 3-phase process to determine the best allocation of resources. Teh fist stage determine and verify the scheduling parameters. In the second stage a estimate a parameter (*α*) that is used to rank UE priority. The final stage designates resources based on the priority rank. This algorithm is compared with other state-of-the-art algorithms by simulation. Although this algorithm is outperformed by other single-metric oriented approaches, the authors conclude that in a multi-metric approach the performance of the proposed algorithm is balanced while sustaining the lowest energy consumption.

In [40] the Energy Aware Scheduling Algorithm (EASA) performance for a 5G Green Network is analyzed. The authors propose an energy-aware scheduling model that considers the characteristics of 5G Green Communications. They present an analytical model to describe the optimization problem. A simulation is conducted to evaluate the model performance. The proposed algorithm uses machine learning to allocate real-time resources based on network conditions and user demand. Simulation results show that there is a reduction in energy consumption while maintaining high performance. The authors conclude that using energy-aware models can contribute to a sustainable environment without affecting performance or incurring operational costs to the grid.

Table 1 presents a summary of the state of the art reviewed.

**Table 1:** State of the art summary.

Work	Network	Technique			
[8]	LTE	Prototype	Model	Simulation	Full Stack
[9]	4G, 5G	Test Bed	✘	✘	✔
[10]	4G, 5G	Test Bed	✘	✘	✔
[11]	WiFi	Test Bed	✘	✘	✔
[12]	5G	Analytical Model	✔	✘	✘
[14]	5G	Analyical Model	✔	✘	✘
[36]	5G	Scheduling Algorithm	✔	✘	✘
[37]	5G	QoS Prediction Algorithm	✔	✘	✘
[38]	5G	Analytical Mode	✔	✘	✘
[39]	4G LTE-A	Scheduling Algorithm	✔	✔	✘
[40]	5G	Scheduling Algorithm	✔	✔	✘

## 3 5G NR cross-layer analytical modeling

**Fig 3:**
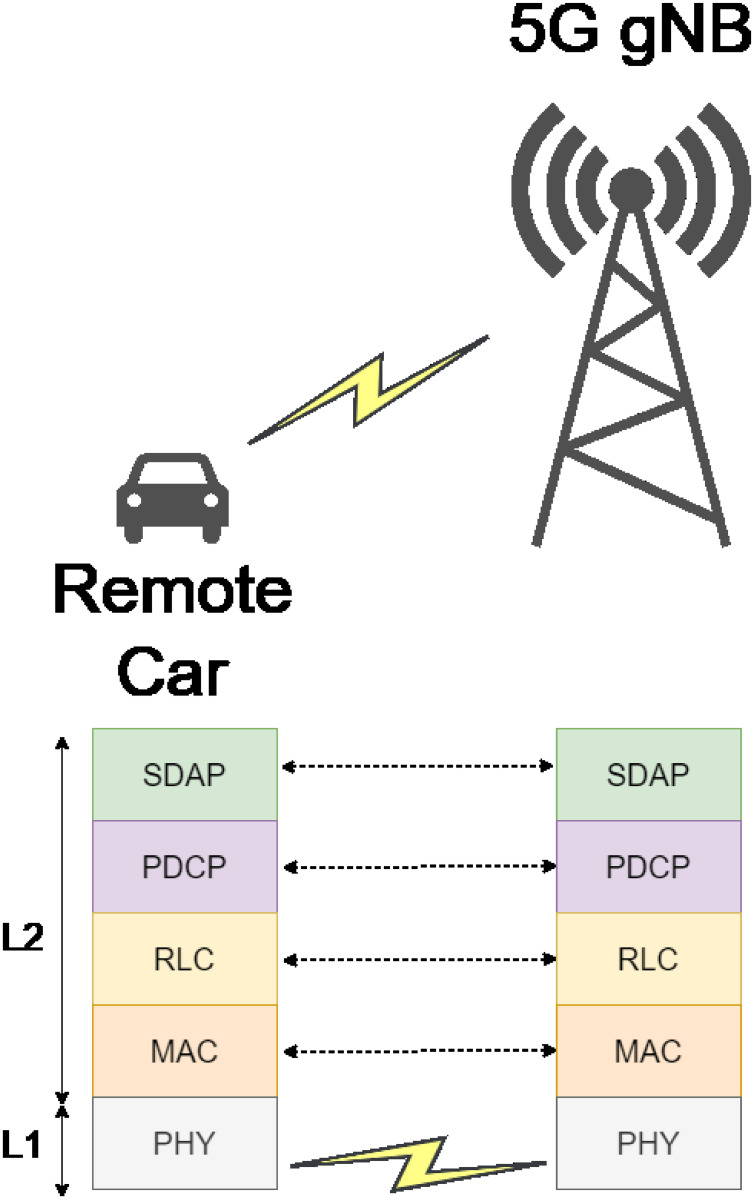
5G new radio protocols.

This paper focuses on 5G NR protocols that enable communication between the user device (*UE*), in this case, the vehicle, and the base station (*gNB*). The analysis is carried out with a protocol layer approach to identify the relevant behaviors of these protocols that influence the communication performance, mainly throughput and latency. For 5G, the radio link enabling protocols are those of layers 1 and 2 of the OSI reference model. These protocols are shown in Fig 3.

It should be noted that the L2 layer is divided into different sub-layers to abstract and simplify the behavior associated with it. The behavior of each of the protocols shown in Fig 3 is based on the 3GPP specifications used to develop the models in this work. Based on the scenario, the one-way delay (*OWD*) of the radio link can be defined as follows:

$$\begin{array}{c} radio_{OWD}=delay_{sdap}+delay_{pdcp}+delay_{rlc}+delay_{mac}+delay_{phy} \end{array}$$ (1)

where *delay*_*x*_ represents the delay introduced by layer *x* with *x* ∈ {*sdap*, *pdcp*, *rlc*, *mac*, *phy*}. Similarly, the performance of the radio link can be defined as:

$$\begin{array}{c} radio_{Th}=min\left(th_{sdap},th_{pdcp},th_{rlc},th_{mac},th_{phy}\right) \end{array}$$ (2)

with *th*_*x*_, the protocol throughput for *x* ∈ {*sdap*, *pdcp*, *rlc*, *mac*, *phy*}. It is necessary to define the performance of the different protocols to evaluate the models presented in Eqs 1 and 2. The remainder of this section describes the appropriate models for each of the protocols considered in radio communication.

### 3.1 Service Data Adaptation Protocol (SDAP) sub-layer

**Fig 4:**
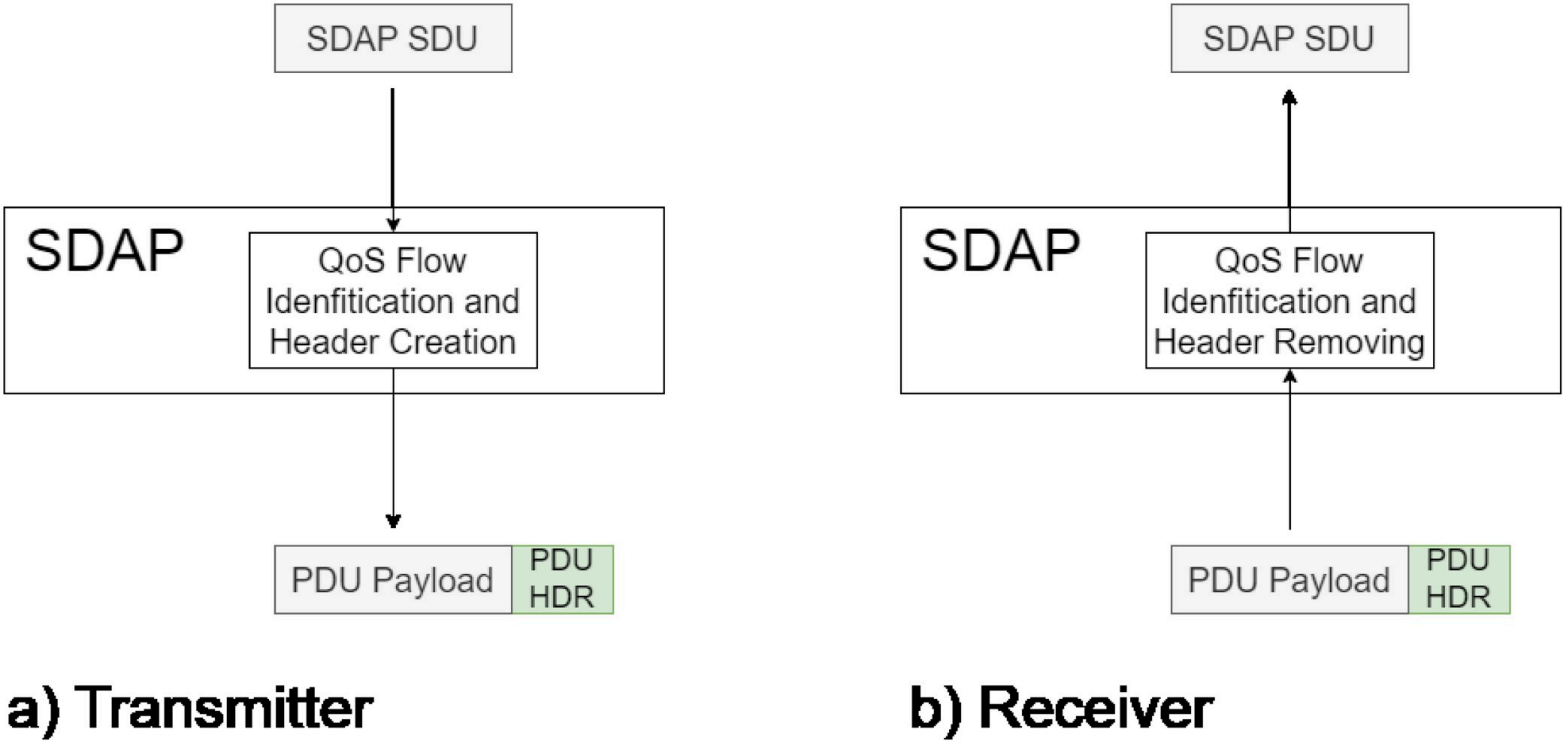
SDAP operation.

The Service Data Adaptation Protocol is the upper sub-layer protocol in L2. The function of SDAP is to manage different levels of QoS through traffic flows associated with each level [15]. This is handled by a QoS Flow Identifier (*QFI*) field included in the SDAP PDU header. On the transmitter side, the SDAP protocol receives IP packets, identifies the type of traffic by checking the appropriate field, and assigns a suitable identifier for this traffic (Fig 4a). After this, the SDAP PDU is generated, which will be retransmitted to the next layer (PDCP) in a virtual channel (radio bearer) on which packets with similar QoS requirements travel.

On the receiver side, SDAP receives a PDU, from which the header is removed to identify its flow through the QFI field and then forwarded to the upper layer (Fig 4b). In the present model, all the packets transmitted by the device are considered to belong to the same class. Therefore, they are all processed similarly and retransmitted to the same virtual channel. Based on this behavior, SDAP can be identified as a store-and-forward element; thus, analyzing it as a queuing system is possible. For UL (Up-Link) on the transmitter side, video packets are assumed to follow a Poisson process. On the other hand, in DL (Down-Link), the packets from the control station arrive quasi-periodically; therefore, their arrival process is deterministic. In both cases, the processing performed by SDAP is considered to have an exponential distribution. Based on this, the behavior of SDAP transmitter can be defined as an M/M/1 system and a D/M/1 system for UL and DL, respectively. Based on queuing theory, the SDAP performance model for the UL direction can be obtained as follows:

$$\begin{array}{c} Th_{tx-SDAP-UL}=min\left(\lambda_{SDAP},\mu_{SDAP}\right) \end{array}$$ (3)

$$\begin{array}{c} Delay_{tx-SDAP-UL}=\frac{1}{\mu_{SDAP}-\lambda_{SDAP}} \end{array}$$ (4)

where λ_*SDAP*_ is the rate at which video packets arrive at the SDAP layer, and *μ*_*SDAP*_ is the rate at which SDAP services incoming packets. For the DL direction, the throughput model is:

$$\begin{array}{c} Th_{tx-SDAP-DL}=min\left(\frac{1}{T},\mu_{SDAP}\right) \end{array}$$ (5)

here, *T* is the period between control packets arrivals. The following equation is used to calculate the time that each packet spends in the SDAP layer:

$$\begin{array}{c} Delay_{tx-SDAP-DL}=\frac{1}{\mu_{SDAP}\left(1-\sigma \right)} \end{array}$$ (6)

where, *σ* is the solution of *σ* = *e*^−*μT*(1−*σ*)^ with the lowest absolute value. Using the Eqs 3–6, SDAP performance can be evaluated in both communication directions. The results of this evaluation are presented in section 4.

### 3.2 Packet Data Convergence Protocol (PDCP) sub-layer

**Fig 5:**
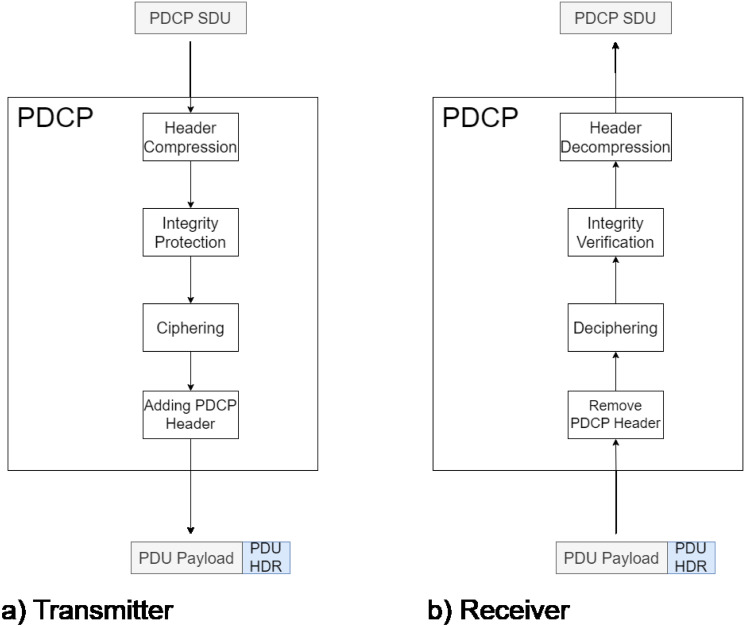
PDCP operation.

The PDCP layer provides security and integrity protection for 5G communications and performs header compression [16]. PDCP functions are depicted in Fig 5. According to [2], the main functionality of header compression is to match 5G voice services with legacy voice services, which have no packet header. This analysis considers only data transmission; therefore, the header compression mechanism is not considered.

On the PDCP, the transmitter side receives SDUs from SDAP. Each SDU is assigned an integrity code called Message Authentication Code (*MAC-I*), then encrypted, and a header and trailer are added to generate the PDCP PDU (See Fig 5a. No header compression is performed). The reverse process is done on the receiver side: first stripping the header, then checking the integrity using the MAC, and decrypting the content before sending it to the upper layer (Fig 5b). Thus, the behavior of PDCP is a process of three sequential service phases. Based on this, PDCP is described as a *M*/*Hypo*_3_/1 queuing system. The geometric matrix approach is used for the analysis of this system. The queuing system has associated a transition matrix with a block structure of the form:

$$\begin{array}{c} Q=[\begin{array}{cccccc} B_{00} & B_{01} & 0 & 0 & 0 & \cdots \\ B_{10} & A_{1} & A_{2} & 0 & 0 & \cdots \\ 0 & A_{0} & A_{1} & A_{2} & 0 & \cdots \\ 0 & 0 & A_{0} & A_{1} & A_{2} & \cdots \\ 0 & 0 & 0 & A_{0} & A_{1} & \cdots \\ 0 & 0 & 0 & 0 & A_{0} & \cdots \\ \vdots & \vdots & \vdots & \vdots & \vdots & \ddots \end{array}] \end{array}$$ (7)

where the component block matrices are:

$$\begin{array}{c} B_{00}=\left[-\lambda \right] \end{array}$$ (8)

$$\begin{array}{c} B_{01}=\left[\lambda \;0\;0\right] \end{array}$$ (9)

$$\begin{array}{c} B_{10}=[\begin{array}{c} 0\\ 0\\ \mu_{3} \end{array}] \end{array}$$ (10)

$$\begin{array}{c} A_{0}=[\begin{array}{ccc} 0 & 0 & 0\\ 0 & 0 & 0\\ \mu_{3} & 0 & 0 \end{array}] \end{array}$$ (11)

$$\begin{array}{c} A_{1}=[\begin{array}{ccc} -(\lambda +\mu_{1}) & \mu_{1} & 0\\ 0 & -(\lambda +\mu_{2}) & \mu_{2}\\ 0 & 0 & -(\lambda +\mu_{3}) \end{array}] \end{array}$$ (12)

$$\begin{array}{c} A_{2}=[\begin{array}{ccc} \lambda & 0 & 0\\ 0 & \lambda & 0\\ 0 & 0 & \lambda \end{array}] \end{array}$$ (13)

In Eqs 8–13, the value λ is the rate at which packets arrive at the PDCP layer and *μ*_1_, *μ*_2_ and *μ*_3_ are the service rates associated to the MAC-I calculation, ciphering and header creation process, respectively.

These equations are associated with the dynamics of the block matrix *Q* (Eq 7), allowing to describe how it transits between its levels. The level directly corresponds to the number of packets in the queue. Specifically, level 0 denotes no packets waiting, level 1 signifies 1 packet, and so on. The matrix in the Eq 8 indicates the rate at which the level 0 is exited. Eq 10 represents the rate at which the level 0 is entered. Similarly, there are matrices that describe the dynamics when *i* > 0 packets are in the queue. In this case, these are square matrices of order *n*, where *n* = 3 is the number of sequential phases that comprise the service process. The matrix *A*_0_ (Eq 11) it is the rate at which a package transits from level *i* to level *i* − 1, which occurs when a package exits the 3rd phase of service (at a *mu*_3_ rate). Matrix *A*_1_ (Eq 12) contains the rates of transitions between the different phases of service. Finally, matrix *A*_2_ is the rate at which level *i* transitions to level *i* + 1, which occurs at λ. Since this transition can occur during any phase of service, *A*_2_ matrix is constructed to represent this.

MAC-I computation and encryption processing rates are associated with the data size. Therefore, these service rates must be estimated for the data sizes of interest in the present analysis.

The *M*/*Hypo*_3_/1 queue transition matrix (Eq 7) has a steady state vector ***π*** that satisfies:

$$\begin{array}{c} \pi Q=0 \end{array}$$ (14)

where the vector ***π*** has the form ***π*** = [***π***_0_ ***π***_1_ ***π***_2_ …]. Here, the element ***π***_*i*_ = [*π*_*i*,1_*π*_*i*,2_*π*_*i*,3_] is a vector associated with the probability that the queue has *i* packets in the queue and the current packet in service is in the *j* ∈ [1, 2, 3] phase of service. The elements of ***π*** are geometrically related by a matrix R such as:

$$\begin{array}{c} \pi_{i}=\pi_{1}R^{i-1} \end{array}$$ (15)

The state of the queue is completely described by the ***π***_0_ and ***π***_1_ vectors and the *R* solution matrix. The work [17] describes the R matrix in a closed form as:

$$\begin{array}{c} R=[\begin{array}{ccc} \frac{\lambda \left(\lambda +\mu_{2}\right)\left(\lambda +\mu_{3}\right)}{\mu_{1}\mu_{2}\mu_{3}} & \frac{\lambda (\lambda +\mu_{3})}{\mu_{2}\mu_{3}} & \frac{\lambda }{\mu_{3}}\\ \frac{\lambda^{2}\left(\lambda +\mu_{2}+\mu_{3}\right)}{\mu_{1}\mu_{2}\mu_{3}} & \frac{\lambda (\lambda +\mu_{3})}{\mu_{2}\mu_{3}} & \frac{\lambda }{\mu_{3}}\\ \frac{\lambda^{2}\left(\lambda +\mu_{2}\right)}{\mu_{1}\mu_{2}\mu_{3}} & \frac{\lambda^{2}}{\mu_{2}\mu_{3}} & \frac{\lambda }{\mu_{3}} \end{array}] \end{array}$$ (16)

The vectors ***π***_0_ and ***π***_1_ are those that satisfy the normalization condition:

$$\begin{array}{c} \pi_{0}+\pi_{1}{\left(I-R\right)}^{-1}e=1 \end{array}$$ (17)

with *I* a 3x3 identity matrix and *e* = [1, 1, 1]^*t*^. Once the vectors ***π***_0_, ***π***_1_, and the matrix *R* are known, the statistics of the queue can be evaluated. The queue throughput is obtained as follows:

$$\begin{array}{c} PDCP_{Th}=min\left(\lambda ,\frac{\mu_{1}\mu_{2}\mu_{3}}{\mu_{2}\mu_{3}+\mu_{1}\mu_{3}+\mu_{1}\mu_{2}}\right) \end{array}$$ (18)

**Fig 6:**
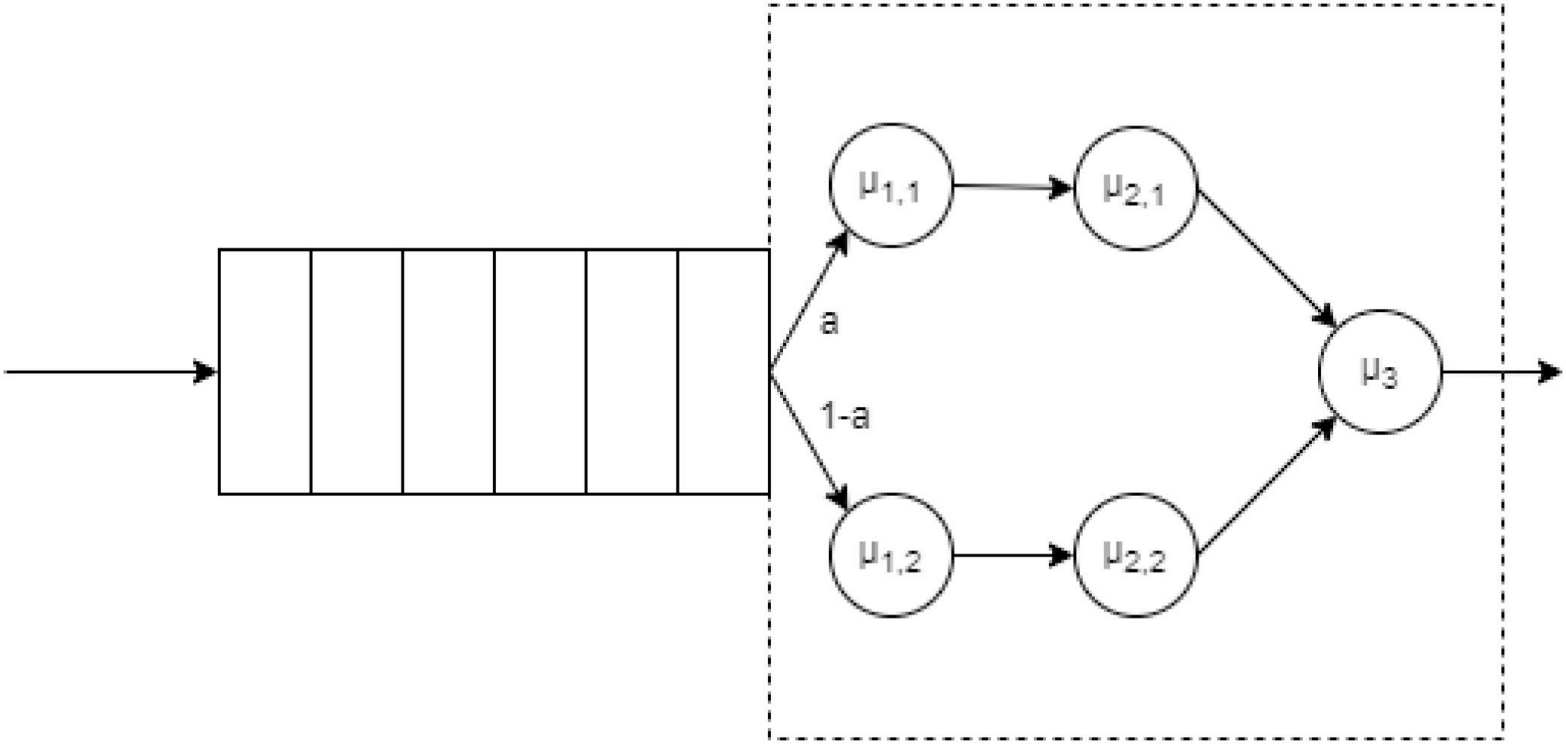
PDCP operation for two packet sizes.

In addition, the delay is defined as:

$$\begin{array}{c} PDCP_{Delay}=\frac{|\pi_{1}{\left(I-R\right)}^{-2}{|}_{1}}{\lambda } \end{array}$$ (19)

where |***x***|_1_ is the 1-norm of vector ***x***. The model presented above considers that all packets arriving at the PDCP layer have the same size. This is the case for transmitting driver control messages (DL directions). However, video streaming has two different packet sizes (one for 1280x720 and the other for 640x480 video). Thus, once the video packets arrive at the PDCP, the processing time of each of these packets must be differentiated. This leads to slightly modifying the approach adopted for the PDCP model. In this case, the service is no longer hypoexponential but follows a sequence of phases of service, as shown in Fig 6. where *a* is the probability that the arriving packet has high-resolution video, *μ*_1,1_ and *μ*_2,1_ are the ciphering and integrity protection rates for high-resolution video packets. While *μ*_1,2_ and *μ*_2,2_ are those rates for standard-resolution video packets. Based on this behavior, it can be identified that the service process is phase type (*PH*). These distributions have an associated rate matrix that guides the transition dynamics between the different phases. Using this matrix, the service process can be characterized, and the performance of the queuing system can be analyzed. For this, a geometric matrix approach is used, similar to that used to analyze the *M*/*Hypo*_3_/1 system presented above. However, unlike this one, there is no explicit solution to find the *R* matrix associated with the system. Based on the Eq 14 it is identified that:

$$\begin{array}{cc} \pi_{0}B_{00}+\pi_{1}B_{10} & =0\\ \pi_{0}B_{01}+\pi_{1}A_{1}+\pi_{2}A_{0} & =0\\ \pi_{i-1}A_{2}+\pi_{i}A_{1}+\pi_{i+1}A_{0} & =0,i=2,3,4,... \end{array}$$ (20)

where:

$$\begin{array}{c} B_{00}=\left[-\lambda \right] \end{array}$$ (21)

$$\begin{array}{c} B_{01}=\left[\left(1-a\right)\lambda \;a\lambda \;0\;0\;0\right] \end{array}$$ (22)

$$\begin{array}{c} B_{10}=[\begin{array}{c} 0\\ 0\\ 0\\ 0\\ \mu_{3} \end{array}] \end{array}$$ (23)

$$\begin{array}{c} A_{0}=[\begin{array}{ccccc} 0 & 0 & 0 & 0 & 0\\ 0 & 0 & 0 & 0 & 0\\ 0 & 0 & 0 & 0 & 0\\ 0 & 0 & 0 & 0 & 0\\ \left(1-a\right)\mu_{3} & a\mu_{3} & 0 & 0 & 0 \end{array}] \end{array}$$ (24)

$$\begin{array}{c} A_{1}=[\begin{array}{ccccc} -(\mu_{1,1}+\lambda ) & 0 & \mu_{1,1} & 0 & 0\\ 0 & -(\mu_{1,2}+\lambda ) & 0 & \mu_{1,2} & 0\\ 0 & 0 & -(\mu_{2,1}+\lambda ) & 0 & \mu_{2,1}\\ 0 & 0 & 0 & -(\mu_{2,2}+\lambda ) & \mu_{2,2}\\ 0 & 0 & 0 & 0 & -(\mu_{3}+\lambda ) \end{array}] \end{array}$$ (25)

Note that states *i* = 2, 3, 4, …. in Eq 20 have the same structure. It is this structure that allows the use of an algorithmic approach. Substituting 15 in 20 yields:

$$\begin{array}{c} \begin{array}{cc} \left(\pi_{1}R^{i-2}\right)A_{2}+\left(\pi_{1}R^{i-1}\right)A_{1}+\left(\pi_{1}R^{i}\right)A_{0} & =0 \end{array} \end{array}$$ (26)

Manipulating Eq 26:

$$\begin{array}{c} \begin{array}{c} \pi_{1}R^{i-2}\left(A_{2}+RA_{1}+R^{2}A_{0}\right)=0 \end{array} \end{array}$$ (27)

Thus, the *R* matrix is the solution of the quadratic matrix equation *A*_2_ + *RA*_1_ + *R*^2^*A*_0_ = **0**. To obtain *R*, it follows that:

$$\begin{array}{cc} A_{2}+RA_{1}+R^{2}A_{0} & =0\\ RA_{1} & =-A_{2}-R^{2}A_{0}\\ R & =-A_{2}A_{1}^{-1}-R^{2}A_{0}A_{1}^{-1} \end{array}$$ (28)

Eq 28 is the base of the algorithm. This is:

1. Initialize $R_{0}=-A_{2}A_{1}^{-1}$
2. Calculate $R_{i}=-A_{2}A_{1}^{-1}-R_{i-1}^{2}A_{0}A_{1}^{-1}$, for *i* = 1, 2, 3, …
3. Repeat step 2 until the value *R* converges, i.e. the difference between *R*_*i*−1_ and *R*_*i*_ is below a threshold.

Note that the delay equation (Eq 19) is still valid. However, the throughput model requires slight modification:

$$\begin{array}{c} PDCP_{Th}=min\left(\lambda ,\frac{1}{PDCP_{st}}\right) \end{array}$$ (29)

where *PDCP*_*st*_ is the mean service time of the PDCP layer. This service time is derived from the PH distribution that describes it. For the presented model it is given by:

$$\begin{array}{c} \frac{\mu_{1,1}\cdot \mu_{1,2}\cdot \mu_{2,1}\cdot \mu_{2,2}\cdot \mu_{3}}{a\left(\mu_{1,2}\mu_{2,2}\right)\mu_{1,1}\mu_{2,1}\mu_{3}+\left(1-a\right)\left(\mu_{1,1}\mu_{2,1}\right)\mu_{1,2}\mu_{2,2}\mu_{3}+\mu_{1,1}\mu_{2,1}\mu_{1,2}\mu_{2,2}} \end{array}$$ (30)

This completes the description of the PDCP model. To evaluate the model, it is necessary to describe the parameters it depends on. λ is given by the packet rates associated with each communication scenario. As stated above, the encryption and integrity protection processes depend on the size of the packet. According to [18], 5G has three algorithm alternatives to perform integrity protection and encryption. The available algorithms are presented in Table 2:

**Table 2:** Algorithms used for integrity protection and ciphering.

Algorithms		
Integrity	Ciphering	Based on
128-NIA1	128-NEA1	SNOW3G
128-NIA2	128-NEA2	AES
128-NIA3	128-NEA3	ZUC

The present study considers the alternative based on AES (i.e., 128-NIA2 and 128-NEA2). The reason for this choice is twofold: it is a non-proprietary option and well documented in the literature. Using a technique like the one presented in [19], the AES algorithms for encryption and integrity protection are implemented in Python and tested to obtain a service time estimate. Testing was performed on a Windows 11 Pro computer with an AMD Ryzen 7 5700G processor with Radeon Graphics @ 3.80 GHz and 32 GB of RAM. Encryption and integrity protection experiments were replicated 100,000 times for each packet size. For UL, these are 467 Bytes and 521 Bytes for packets transmitting 640x480 and 1280x720 video, respectively. For the DL case, considering that there are 50 objects in the vehicle [5] and that 32-bit commands are sent to each of them, the control packets are 200 bytes. This value is within the margins considered in [5, 20] for remote driving applications. The average encryption and integrity protections times presented in Table 3 are obtained from these experiments.

**Table 3:** Average ciphering and integrity protection times for the different packet sizes.

	200 B	467 B	520 B
Ciphering	4.2107E-06 s	4.1865E-06 s	ñ4.3196E-06 s
Integrity	8.7660E-6 s	8.9776E-06 s	9.2588E-06 s

Using the average times presented in Table 3, processing rates for encryption (*μ*_1_) and integrity protection (*μ*_2_) are estimated. Based on these values, the evaluation presented in Section 4 is carried out.

### 3.3 Radio Link Control (RLC) sub-layer

**Fig 7:**
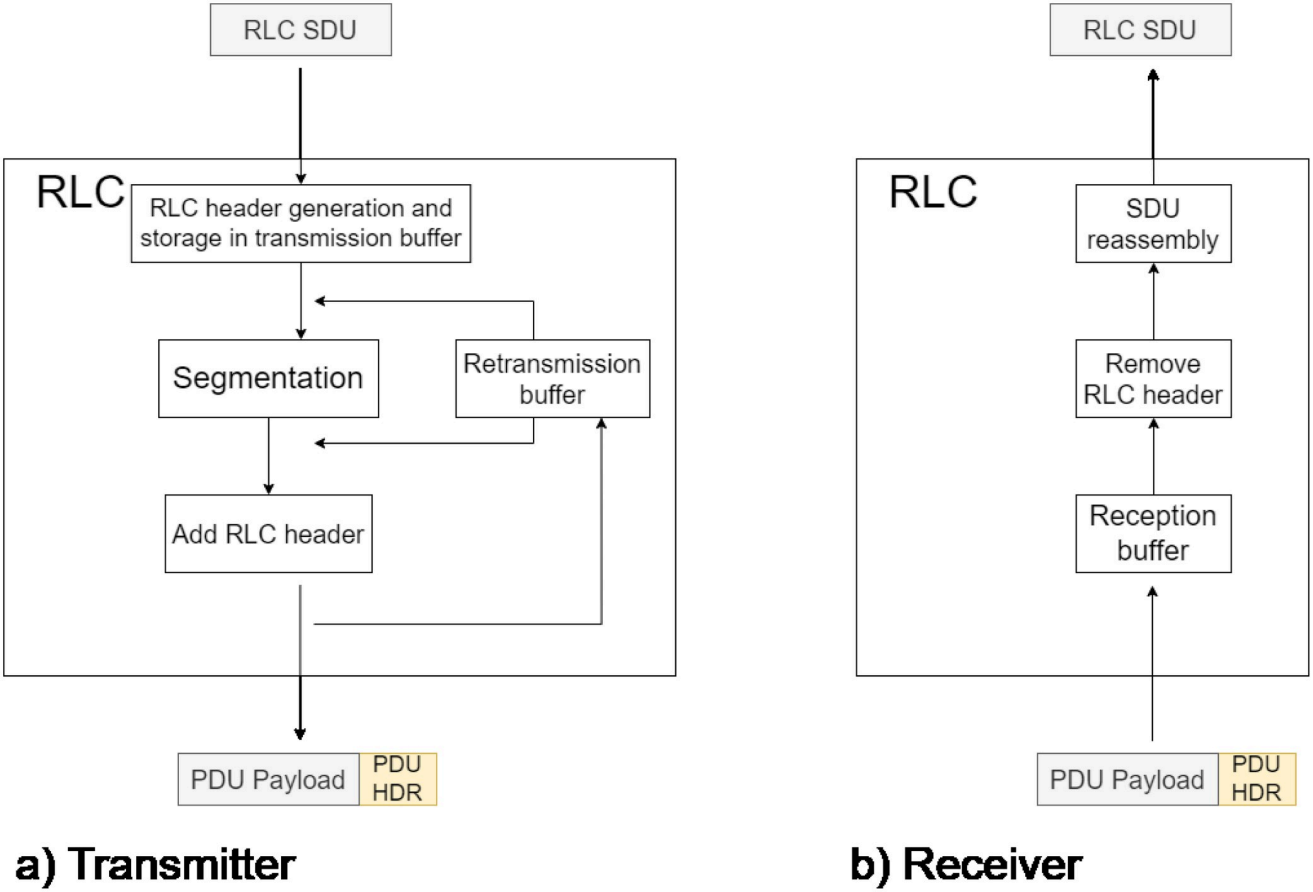
RLC AM operation.

The RLC sublayer’s primary function is transmitting information in one of the three modes of operation available to it [21]. Those modes are Transparent Mode (*TM*), Unacknowledged Mode (*UM*), and Acknowledged Mode (*AM*). The two first operation modes are oriented to Control Plane message transmission. The last one is used for User Plane messages. The RLC AM operation is depicted in Fig 7.

**Fig 8:**
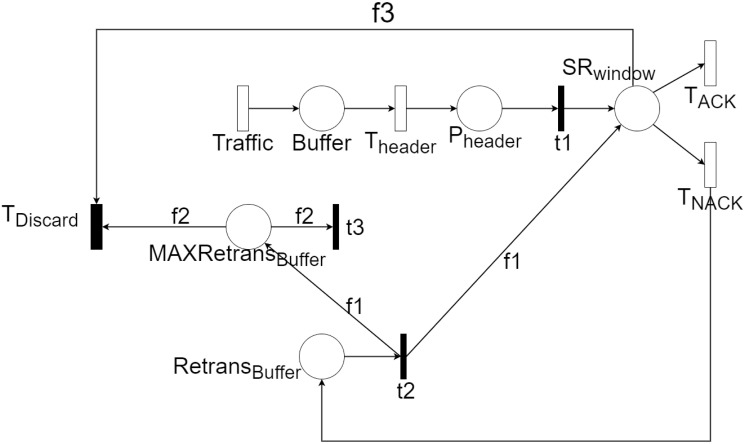
RLC Stochastic Reward Net.

On the transmitter side, the RLC sublayer receives SDUs from the PDCP layer. Each SDU is assigned a header and then forwarded using an Automatic Repeat Request (*ARQ*) re-transmission mechanism (Fig 7a). RLC considers possible packet fragmentation if a large amount of data is received that cannot be transmitted in a single PDU. For the present study, it is assumed that fragmentation is not necessary. On the receiver side, the packets are received, and if no error occurs, the header is stripped, assembled in case of fragmentation, and sent to the next layer (Fig 7b). Due to its behavior, it is challenging to abstract RLC AM as a queuing system. Due to its behavior, it can be challenging to outline RLC AM as a queuing system. Hence, we looked for a different formalism to describe the AM RLC model. Inspired by [22], we model RLC AM as a Stochastic Reward Net (*SRN*). A SRN is a variant of stochastic Petri nets that allows the evaluation of different performance metrics by assigning reward functions. The SRN used to model RLC AM is presented in Fig 8.

Regarding the SRN, the packets are generated with the activation of the *Traffic* timed transition and are stored in the place of the *Buffer*. Once here, a header is added to them at a rate given by *T*_*header*_ and is stored in the *P*_*header*_ location. They are then transmitted (through the immediate transition *t*1) to the *SR*_*window*_ site if there is available room in the transmission window. This is where the ARQ mechanism begins. Packets are correctly transmitted with a rate given by the *T*_*ACK*_ transition. Erroneous transmissions occur at a rate defined by *T*_*NACK*_. Each time this transition fires, a copy of the message is placed in the *Retrans*_*buffer*_ place. Once all packets in *SR*_*window*_ have been attempted to be transmitted, packets in *Retrans*_*buffer*_ are transferred back to *SR*_*window*_ for re-transmission through transition *t*2. This occurs if the maximum number of re-transmissions allowed for each packet has yet to be reached. The *MAXRetrans*_*buffer*_ place controls the number of re-transmissions. Each time a packet is placed in *SR*_*windows*_ for re-transmission (t2 firing), its re-transmission counter in *MAXRetrans*_*buffer*_ is incremented. If the packet is successfully transmitted before the maximum number of re-transmissions, the *t*3 transition resets the *MAXRetrans*_*buffer*_ counter. If a packet’s maximum number of re-transmissions is reached, the packet is removed from the *SR*_*window*_ place, and the *MAXRetrans*_*buffer*_ counter is reset. These two actions are performed by firing the *T*_*discard*_ transition. RLC AM performance analysis is performed using specialized tools for Petri nets. The SHARPE [23] tool is used for the present work. With the Sharpe tool, the relevant performance metrics for the model can be estimated. SHARPE analyzes the Markov chains associated with the Petri net to calculate the performance metrics. In this chain, the states are made up of the Petri net markings. The transitions between these states are associated with the firing of the timed transitions. By analyzing the matrix associated with the Markov chain, the transient and stationary behavior of the chain can be identified. Performance metrics are derived from the steady state, for example, the average number of tokens at each site or the average rate at which each transition is triggered.

Using this tool the throughput of the SRN RLC AM model can found to be:

$$\begin{array}{c} RLC_{Th}=\tau_{T_{ACK}} \end{array}$$ (31)

where *τ*_*T*_ is the mean firing rate of transition *X*. For obtaining the RLC delay, Little’s rule is used at each SRN place. Thus, the RLC delay is:

$$\begin{array}{c} RLC_{Delay}=\frac{\#(Buffer)}{\tau_{Traffic}}+\frac{\#(SR_{window})}{\eta_{T_{header}}}+\frac{\#(Retrans_{Buffer})}{\tau_{T_{NACK}}}+\frac{1}{\mu_{TACK}} \end{array}$$ (32)

here, #(*P*) is the mean number of tokens (packets) in place *P*, and *μ*_*TACK*_ is the rate at which the *TACK* transition fires when there are packets in the *SR*_*window*_ site. Using the Eqs 31 and 32, and the Sharpe tool, it is possible to evaluate the performance of RLC AM. The results of this evaluation are presented in the following section.

### 3.4 Medium Access Control (MAC) sub-layer

The next sub-layer to analyze is the MAC layer. The primary services the MAC sublayer provides are data transfer and radio resource allocation [24]. On the transmitter side, the data transfer is performed by generating MAC PDUs by assigning headers and appropriately formatting the data before sending it to the physical (PHY) layer. On the receiver side, MAC receives the data from the PHY layer and tries to retrieve the data from the packet, and if this process is successful, it is transferred to the next layer. The MAC layer implements mechanisms for reliable transmissions. These include HARQ re-transmission or K-Repetitions mechanisms. In the first, feedback messages are used to control the re-transmission of packets that were erroneously received by the receiver. The package and the *K* − 1 copies are transmitted simultaneously in the second one. This increases the probability that the receiver can decode the data from one of the *K* copies sent. Concerning radio resource allocation, the scheduling mechanisms available in 5G fall into two main categories: dynamic scheduling and semi-static scheduling. In dynamic scheduling, requesting a resource each time a packet is transmitted is necessary, which results in delay and overhead due to the exchange of control messages. For semi-static scheduling, the resources that devices can use for transmitting are pre-allocated. This reduces latency and overhead at the cost of contending for resources. Either way, how the radio resources are structured influences the behavior of the scheduling mechanisms. For the present paper, the issue of scheduling is considered to be solved by using a semi-static mechanism. This means that every time there is a transmission, the device has already determined the resources that will be used to transmit.

**Fig 9:**
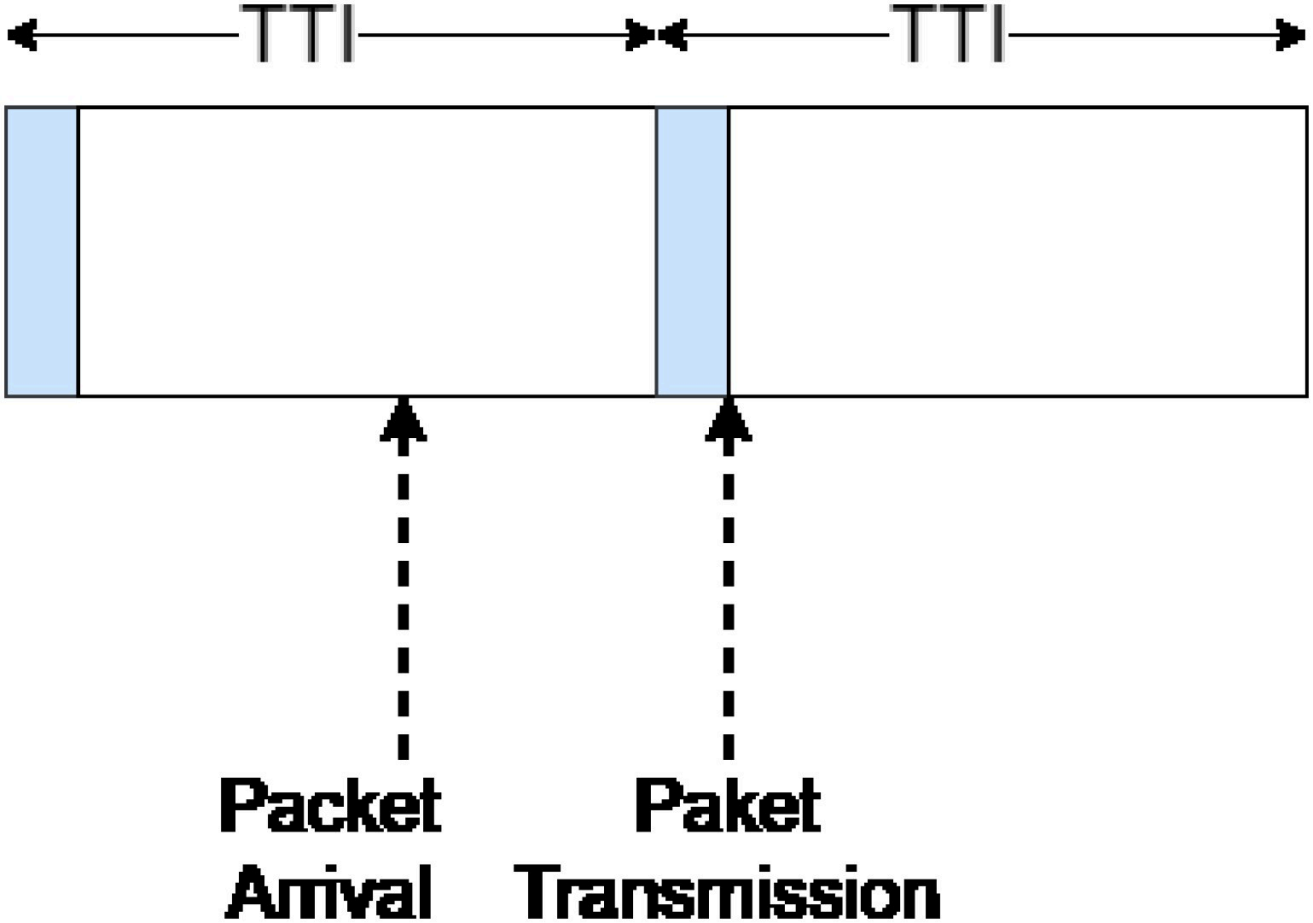
Transmission of the packet in the next transmission opportunity.

Re-transmission mechanisms (if applicable) and scheduling affect MAC behavior and performance. For this research, it is considered that there are no re-transmissions. This is because the messages exchanged (detected video/data and control commands) depend on the context of the vehicle, which changes very quickly. Because of this, messages are short-lived; therefore, it is not worth using resources to re-transmit a message. Also, remote driving packets are supposed to have priority; therefore, resources are pre-allocated to support them. Also, the resources are considered always available to transmit remote drive packets. In other words, the packet will be sent at the next available transmission opportunity; see Fig 9.

This priority assumption can be supported by mechanisms developed for 5G, such as network slicing or preemption techniques. Network slicing generates virtual networks where resources are defined for the different services (for example, eMBB, URLLC, and mMTC). be assigned to each segment to meet the QoS requirements of each service [25]. Regarding the pre-emption methods, puncture techniques are worth mentioning. For instance, the work [26] introduces a resource allocation mechanism in which the resources allocated to eMBB traffic can be punctured (removed) and replaced with traffic data with more stringent requirements, URLLC. Using any of these techniques can guarantee resources to transmit remote drive packets.

Based on these assumptions, MAC behavior is described as *store-and-forward*; where packets arrive, a header is generated for each of them and forwarded at the appropriate time. The MAC headers are created at a rate *μ*_*MAC*_. The time between when the packet is ready for transmission and when it is transmitted is called frame *τ*_*fra*_, whose value is in the range [0, *TTI*]. *TTI* stands for *Transmission Time Interval*, the time resources are allocated in 5G. Based on this, the MAC layer performance model is described as follows:

$$\begin{array}{c} MAC_{Th}=min\left(\lambda_{MAC},\mu_{MAC}\right) \end{array}$$ (33)

here, λ_*MAC*_ is the rate at which the packets arrive at the MAC layer. The mean MAC delay is estimated using the following equation:

$$\begin{array}{c} MAC_{Delay}=\frac{1}{\mu_{MAC}-\lambda_{MAC}}+\frac{TTI}{2} \end{array}$$ (34)

The first component of Eq 34 refers to the delay of the header creation process, and the second to the average frame alignment time.

Specifically, this work considers the K-Repetitions technique. This technique sends *K* copies of the data in a single “shot”. The receiver can use these K copies to try to recover the data. K-Repetitions is generally associated with Grant-Free mechanisms. In this case, it is necessary to consider not only delay but the other component of URLLC, reliability. To meet URLLC requirements for V2X scenarios, i.e., 3-10 ms with reliability of 0.99995, it is necessary to consider the different probabilities that correct data detection will not occur. As presented in ref, to describe the performance of Grant-Free K-Repeats, it is necessary to define the probability that the transmission is detected and the probability that each K-Repetition is correctly received. In [27, Chapter 16], a Grant-Free reliability model with K = 2 repetitions is presented. Using a similar analysis, a model for a K-Repetitions scheme can be defined as:

relK=1-(pmissk+(∑i=0K-1(Ki)(∏j=1ipHARQj)(1-pmiss)k-ipmissi)) (35)

where *p*_*miss*_ is the probability that the transmission pilot is undetected, *p*_*HARQX*_ is the probability that the data cannot be recovered by combining the redundancy of *X* packets, and *p*_*e*_(*p*_*HARQ*1_) is the probability that any of the single K repetitions will be received incorrectly. Using Eq 35 the reliability for a *K* = 4 repetitions is:

$$\begin{array}{c} \begin{array}{cc} rel_{K=4} & =p_{HARQ2}p_{HARQ3}p_{HARQ4}p_{e}{\left(1-p_{miss}\right)}^{4}\\ & +4p_{HARQ2}p_{HARQ3}p_{e}p_{miss}{\left(1-p_{miss}\right)}^{3}\\ & +6p_{HARQ2}p_{e}p_{miss}^{2}{\left(1-p_{miss}\right)}^{2}\\ & +4p_{e}p_{miss}^{3}\left(1-p_{miss}\right)\\ & +p_{miss}^{4} \end{array} \end{array}$$ (36)

In this case, the packet delay depends on which data packet is received correctly. To estimate this delay, the following equation is proposed:

$$\begin{array}{c} Delay_{K=4}=\sum_{i=1}^{4}\left(\frac{\left(rel_{K=i}-rel_{K=(i-1)}\right)}{rel_{K=4}}\right)\cdot i\cdot TI \end{array}$$ (37)

where (*rel*_*K* = *i*_ − *rel*_*K* = (*i*−1)_) represents the probability that the packet was successfully received at the *i*−*th* attempt, and *TI* refers to the packet transmission interval. In addition to the K-repeat delay (Eq 37, the MAC packet header generation time must be considered. Thus, the MAC layer delay operating with Grant-Free K-Repeats is defined as:

$$\begin{array}{c} MAC_{Delay\_GF}=\frac{1}{\mu_{MAC}-\lambda_{MAc}}+T_{align}+Delay_{K=4} \end{array}$$ (38)

where the first component refers to the queue delay, the second indicates the mean alignment time a packet waits before its transmission. This time is calculated assuming the packet will be transmitted in the RB following its creation. This packet creation can occur at any instant; therefore, the alignment time is uniformly distributed over the interval [0, *T*_*symbol*_]. Finally, the K-Repetition delay is the third component of Eq 38.

The Throughput of the MAC operating the K-Repetitions scheme can be derived based on the *T*_*shot*_ interval; in this case, this is:

$$\begin{array}{c} MAC_{Th\_GF}=min\left(\lambda_{URLLC},\frac{1}{T_{shot}}\right) \end{array}$$ (39)

where, λ_*URLLC*_ is the URLLC packets arrival rate. The value of 1/*T*_*shot*_ represents the maximum number of K-repetition shots that can be transmitted. This time is defined as:

$$\begin{array}{c} T_{shot}=K\cdot TI \end{array}$$ (40)

Using the MAC model Eqs (33) and (34), the performance of the MAC layer is evaluated, and the results are presented in Section 4. Similarly, using the Eqs 37 and 39, the performance of the K-Repetitions mechanism for URLLC is evaluated.

### 3.5 Physical (PHY) layer

The PHY layer is where the packets, converted into signals, travel through the channel from the sender to the receiver. The performance of the PHY layer depends mainly on the chosen parameters (for example, bandwidth, coding and modulation order, etc.) and the channel conditions. The present study considers that the network operates in good channel conditions (best-case scenario). Based on this, the maximum PHY throughput model described by 3GPP in [28] is used. This model is:

$$\begin{array}{c} PHY_{Th}=\sum_{j=1}^{J}\left(v_{layer}^{\left(j\right)}\cdot Q_{m}^{\left(j\right)}\cdot R_{max}\frac{N_{PRB}^{BW^{\left(j\right)}}\cdot 12}{T_{s}^{\mu }}\cdot \left(1-OH^{\left(j\right)}\right)\right)bps \end{array}$$ (41)

where:

- *J* is the number of aggregated component carriers in a band or combination. It has a value of 16 for 5G NR UL and DL in [29].
- *R*_*max*_ is 948/1024, the maximum number of Low-Density Parity Check (*LDPC*) obtained from [30].
- For the *j* − *th* Component Carrier (CC):
  - $v_{Layers}^{\left(i\right)}$ is the maximum number of supported layers given by the higher layer parameter maxNumberMIMO-LayersPDSCH for downlink and a maximum of higher layer parameters maxNumberMIMO-LayersCB-PUSCH and maxNumberMIMO-LayersNonCB-PUSCH for uplink. Options for this variable values for UL and DL are specified in [29, 31].
  - $Q_{m}^{\left(j\right)}$ is the maximum supported modulation order given by the higher layer parameter supportedModulationOrderDL for downlink and the higher layer parameter supportedModulationOrderUL for uplink. Values for QPSK, 16QAM, 64QAM, and 256QAM for UL and DL can be obtained in [32].
  - *f*^(*j*)^ is the scaling factor given by the higher layer parameter scalingFactor and can take the values 1, 0.8, 0.75, and 0.4.
  - *μ* is the numerology [32].
  - $T_{s}^{\mu }$ is the average OFDM symbol duration in a subframe for numerology *μ*. Assuming a normal cyclic prefix the value is $T_{s}^{\mu }=\frac{10^{-3}}{14.2^{\mu }}$
  - $N_{PRB}^{BW^{\left(j\right)}\mu }$ is the maximum RB allocation in the bandwidth *BW*^(*j*)^ with numerology *μ* as defined in [33, 34]. *BW*^(*j*)^ is the UE maximum supported bandwidth in a specific band or band combination.
  - *OH*^(*j*)^ is overhead and can take one of the following values:
    1. 0.14 for DL in frequency range FR1
    2. 0.18 for DL in frequency range FR2
    3. 0.08 for UL in frequency range FR1
    4. 0.10 for UL in frequency range FR2

The maximum throughput limit for a given set of parameters is established using the model presented in Eq 41. In a real scenario, the actual throughput is expected to be less than or equal to this value. Regarding the delay, considering different components, such as propagation time, transmission time, and other processing times, is necessary. The model describes the delay of the PHY layer:

$$\begin{array}{c} PHY_{Delay}=\tau_{proc_{tx}}+\tau_{tx}+\tau_{prop}+\tau_{proc_{rx}} \end{array}$$ (42)

where $\tau_{proc_{tx}}$ and $\tau_{proc_{rx}}$ are the processing time at the transmitter and receiver side, respectively; *τ*_*tx*_ is the transmission delay, and *τ*_*prop*_ is the propagation time. The transmission delay is related to the radio resources required to transmit the data and is associated with the configuration parameters. The propagation delay depends on the distance between the transmitter and the receiver; thus, it is limited by the size of the 5G cell. Finally, it is necessary to describe the processing times ($\tau_{proc_{tx}}$ and $\tau_{proc_{rx}}$). Following [12], these values are given by $tau_{proc_{tx}}=T_{proc,2}/2$ and $\tau_{prox_{rx}}=T_{proc,1}/2$. Here, *T*_*proc*,1_ is the UE Physical Downlink Shared Channel (PDSCH) processing procedure time, and *T*_*proc*,2_ is the Physical Uplink Shared Channel (PUSCH) preparation procedure time. Both depend on the device’s processing capabilities and are defined in [30]. The performance of the PHY layer can now be evaluated using Eqs 41 and 42.

The following section evaluates all the models developed in this section, considering a V2X scenario for the remote driving application.

## 4 Model results

The present section uses the models presented in section 3 to evaluate the performance of 5G radio communication for a remote driving usage scenario. First, the remote driving scenario is described. Then, the configuration parameters for 5G NR are described. Finally, the performance evaluation results of the different 5G NR protocols operating with the defined parameters are presented.

### 4.1 Evaluation scenario

The deployment scenario is shown in Fig 2b. This scenario presents a deployment in MEC. Therefore, communication occurs only on the 5G radio network and is mainly supported by 5G NR protocols.

**Fig 10:**
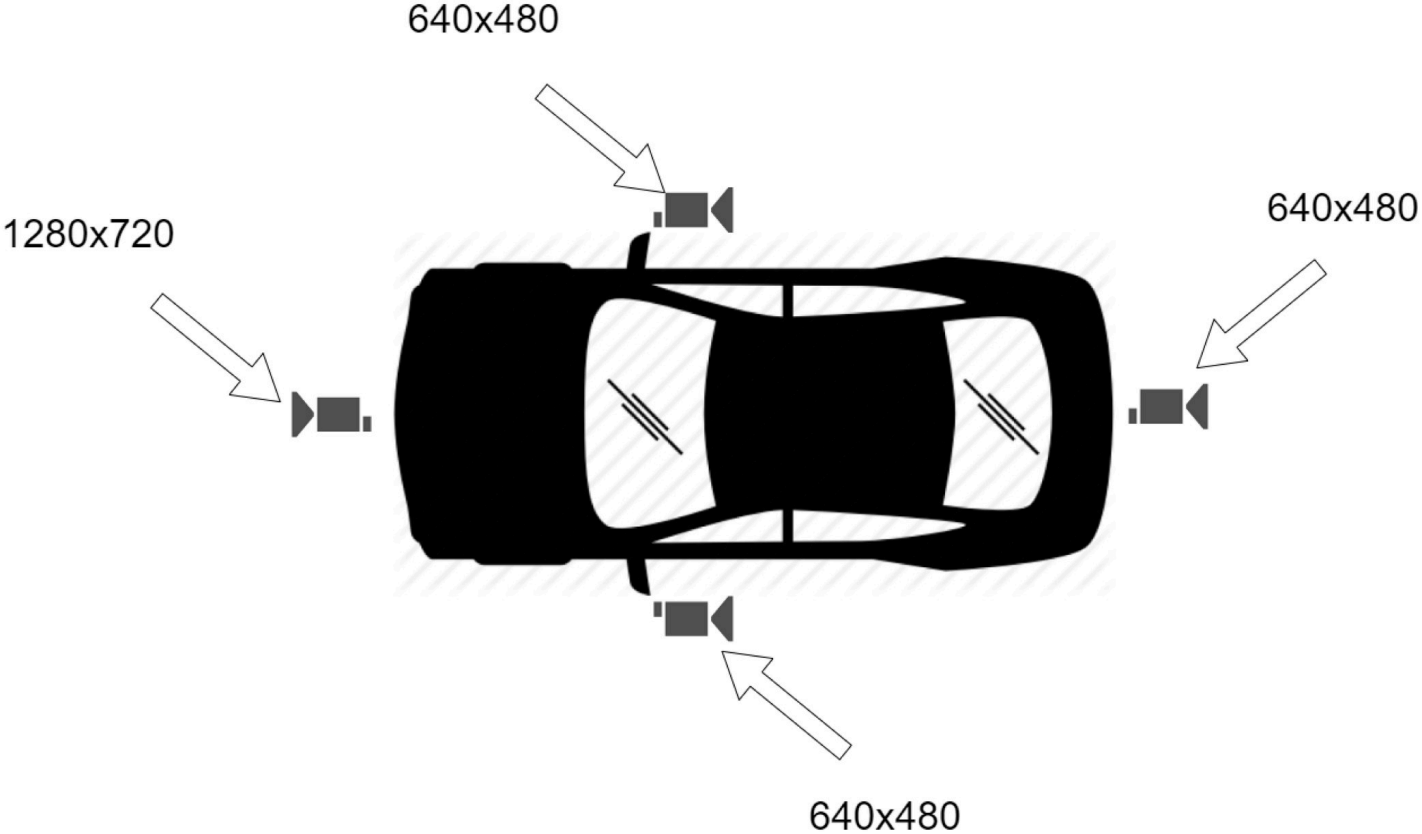
Remote driving study scenario.

As mentioned above, the remote driving application requires vehicle status data, mainly video, to be transmitted to the remote driver. At the same time, the driver is required to send control commands. These transmissions are identified as Uplink when their origin is the vehicle and Downlink when the remote driver generates them; therefore, its source is the gNB for the radio link. In the case of UL, the traffic depends on the resolution, encoding, and other parameters associated with the video. This transmission requires higher bandwidth and is one of the critical points for the definition of remote driving applications. For the proposed scenario, it is considered that the vehicle has 4 cameras that transmit the vision of the vehicle’s surroundings. This is depicted in Fig 10.

While some references mention that are needed between 25 and 32 Mbps to stream video from the vehicle’s four cameras,e.g., [5] states that 8 Mbps is required to stream a high-resolution video stream, other works present real measurements where this requirement is less demanding. For example, in [10], the authors report that 22kBps (176kbps) and 67kBps (536kbps) are required to stream videos in 640x480 and 1280x720 resolution, respectively. According to their measurements, the videos are transmitted at a mean rate of 47.3 and 129.1 packets/s. The present study considers this last scenario for the video transmission evaluation. Considering that the cameras are independent, the total traffic transmitted in UL is the sum of the traffic of each camera. Thus, the UL traffic is defined as:

$$\begin{array}{c} \begin{array}{c} \lambda_{UL}=\lambda_{front}+\lambda_{rear}+\lambda_{left}+\lambda_{right}\\ =129.1+47.3+47.3+47.3=271packets/s \end{array} \end{array}$$ (43)

where, λ_*UL*_ is the UL traffic rate and λ_*front*_,λ_*rear*_,λ_*left*_ and λ_*right*_ are the traffic rates associated with the front, rear, left and right cameras, respectively. For DL transmission, it is considered that the control commands originated by the driver are transmitted periodically. This is done to ensure that only a few orders during the transmission period of the lost packet are affected in case of packet loss. Typically, these transmissions are defined to occur several times per second; for example, 5GAA [5] states that 50 transmissions per second (20ms), and [20] mentions that they occur every 10ms (100 times per second). The model evaluation considers both scenarios, referred to as *DL*_*A*_ and *DL*_*B*_.

The 5G network configuration parameters are based on those presented in [12], oriented to 5G V2X scenarios. These are summarized in Table 4:

**Table 4:** 5G NR configuration parameters.

Parameters	
BW	20 MHz
CP	Normal Cyclic Prefix (NCP)
Slot	14 OFDM Symbols (full-slot)
NRB	51 RB
Q (Modulation)	8
R (Coding Rate)	0.8643
*μ*	1 (Numerology)
SCS	15⋅2^*μ*^*kHz* = 30*kHz*
v	2 MIMO-Layers

Here, *BW* is the bandwidth used for the 5G network. *CP* is the employed cyclic prefix; its value is 14 meaning a normal prefix. *Slot* is the size of the *TTI* given in OFDM symbols. *NRB* are the Resource Blocks (RB) available for the given bandwidth and specific numerology; it is provided by [33]. The numerology *μ* with *μ* ∈ {0, 1, 2, 3, 4} defines the sub-carrier spacing (SCS). Finally, *v* determines the maximum MIMO (Multiple-Input, Multiple-Output) layers. For this analysis, we consider a maximum of two MIMO layers.

### 4.2 SDAP model evaluation

The SDAP performance in the UL direction on the transmitter side is calculated using Eqs 3 and 4. These performance values for the interest traffic rate (271*packets*/*s*) are:

$$Th_{SDAP\_UL}=271packets/s$$

$$Delay_{SDAP\_UL}=2.000e-8s$$

For the DL direction performance equations, 5 and 6 are employed. In this scenario, the relevant traffic rates are *DL*_*A*_ = 50*packets*/*s*[5] and *DL*_*B*_ = 100*packets*/*s*[20]. The performance values for these relevant rates are

$$Th_{SDAP\_DL_{A}}=50packets/s$$

$$Delay_{SDAP\_DL_{A}}=2.000e-8s$$

for the former rate, and,

$$Th_{SDAP\_DL_{B}}=100packets/s$$

$$Delay_{SDAP\_DL_{B}}=2.000e-8s$$

for the latter.

### 4.3 PDCP model evaluation

The PDCP performance obtained using the models described in section 3.2 are presented below. As PDCP performance is affected by the size of the transmitted packet, it is necessary to perform a specific evaluation for each of these packet sizes. In this case, the UL traffic rate of 271*packets*/*s* is composed of 129.1*packets*/*s* containing HD video and 141.9*packets*/*s* with SD video. Based on this, 47.64% of the arriving packets contain HD, and 52.36% contains SD video. Using these values, the following performance results are obtained:

$$Th_{PDCP\_UL}=271packets/s$$

$$Delay_{PDCP\_UL}=3.3582e-5$$

On the other hand, the performance metrics related to the DL relevant rates are

$$Th_{PDCP\_DL_{A}}=50packets/s$$

$$Delay_{PDCP\_DL_{A}}=3.2945e-5$$

for the *DL*_*A*_ rate, and they are

$$Th_{PDCP\_DL_{B}}=100packets/s$$

$$Delay_{PDCP\_DL_{B}}=3.2945e-5.$$

for the *DL*_*B*_ rate.

### 4.4 RLC model evaluation

The performance of the RLC layer is evaluated using the model presented in section 3.3. As mentioned above, the RLC model is evaluated using the SHARPE tool. The model is configured using the parameters in Tables 5–7:

**Table 5:** Timed transition configuration parameters.

Transition	Value
Traffic	*PDCP*_*Th*_
*T*_*header*_	0.02^−1^*packets*/*ms*
*T*_*ACK*_	*p*/0.01*packet*/*ms*
*T*_*NACK*_	(1 − *p*)/0.01*packets*/*ms*

**Table 6:** Parameters for activating immediate transitions.

Transition	Activation Condition
*t*1	#(*SR*_*W*_*indow*) + #(*Retrans*_*B*_*uffer*)) < *window*_*size*
*t*2	#(*SR*_*W*_*indow*) == 0
*T*_*Discard*_	#(*MaxRetrans*_*Buffer*_)<*max*_*ret*
*t*_3_	#(*MaxRetrans*_*Buffer*_)<*max*_*ret*)&(#(*SR*_*W*_*indow*) == 0) &(#(*Retrans*_*Buffer*_) == 0

**Table 7:** Constant parameters.

Parameter	Value
*p*	0.97
*window*_*size*	10
*max*_*ret*	20

Where *p* is the packet success transmission, *windows*_*size* is the size of the ARQ mechanism and *max*_*ret* is the maximum number of repetitions allowed for each packet Using it, the SRN associated with RLC (Fig 8) is defined, and the rewards associated with the throughput and latency metrics are evaluated. The results of this evaluation are presented below. First, results associated with UL streams containing video are displayed. The results associated with this stream are

$$Th_{RLC\_UL}=271packets/s$$

$$Delay_{RLC\_UL}=4.0309e-8s$$

The DL directions performance metrics are

$$Th_{RLC\_DL_{A}}=50packets/s$$

$$Delay_{RLC\_DL_{A}}=1.0e-8s$$

and,

$$Th_{RLC\_DL_{B}}=100packets/s$$

$$Delay_{RLC\_DL_{B}}=1.0e-8s$$

for the *DL*_*A*_ rate and *DL*_*B*_ rate, respectively.

### 4.5 MAC model evaluation

The next sub-layer to analyze is the MAC. In this case, the results presented are based on the model developed in Section 3.4. The MAC layer behavior depends on the data size, which determines the resources necessary for the transmission. The resources needed to transmit each packet type are determined using Table 4 parameters. Some codes developed in [12] are used for the needed resource calculation. The authors made these codes available in [35]. Table 8 presents the results for each packet type. These results include the Transport Block Size (*TBS*), i.e., the number of bits to be transmitted and the RB required.

**Table 8:** Resource required for the packet transmissions.

Packet Content	TBS	RBs
Video (640x480)	4224 bits	2 RB
Video (1280x720)	6272 bits	3 RB
Control Commands	2088 bits	1 RB

Based on the results presented in Table 8, the performance analysis of the MAC layer is performed. It highlights the fact that the assumed network conditions are resulting in the amount of resources needed being low. Furthermore, this supports the assumption that the package will be broadcast at the next available transmission opportunity. The results obtained from the performance evaluation of the MAC layer for the UL direction are

$$Th_{MAC\_UL}=271packets/s$$

$$Delay_{MAC\_UL}=2.5002e-8s$$

For the DL direction, the control packets are sent using URLLC. Based on the Configuration Parameters shown in Table 4, these packets can be transmitted using a single RB. The RB duration is defined as *T*_*symbol*_. This time is defined as:

$$\begin{array}{c} T_{symbol}=\frac{10^{-3}}{14\cdot 2^{\mu }} \end{array}$$ (44)

Using the definition of *T*_*symbol*_ to calculate the value of *TI* defined in Eq 39 for a shot of 4*repetitions* is *TI* = 4 ⋅ *T*_*symbol*_ = 40^−1^/14 ⋅ 2^*μ*^. The rest of the parameters described in the equation are defined in Table 9.

**Table 9:** Values for the error probabilities parameters in Eq 35.

Parameter	Value
*p*_*miss*_	< = 0.006
*p*_*e*_	0.1
*p*_*HARQ*2_	0.03
*p*_*HARQ*3_	0.04
*p*_*HARQ*4_	0.05

Referring to Table 9, the value of *p*_*e*_ refers to the probability that a packet is received with an error. It is derived from the Block Error Rate (BLER). The values of *p*_*HARQX*_, *X* ∈ {2, 3, 4}, refer to the probability that a packet cannot be retrieved using the redundancy mechanism with *X* packages. Finally, the value of *p*_*miss*_ <= 0.006 is chosen to ensure that the reliability is as required by URLLC. These pilots can be transmitted over the control channels, making the transmission more reliable. Now that these parameters have been set, it is possible to use Eqs 37 and 39 to obtain the performance of the MAC layer operating with URLLC. In particular, the MAC downlink performance for dataflows *DL*_*A*_ and *DL*_*B*_ can be calculated as:

$$Th_{MAC\_DL_{A}}=50packets/s$$

$$Delay_{MAC\_DL_{A}}=2.8765e-5s$$

$$Th_{MAC\_DL_{B}}=100packets/s$$

$$Delay_{MAC\_DL_{B}}=2.8765e-5s$$

### 4.6 PHY model evaluation

Finally, the performance of the PHY layer is analyzed. The evaluation is based on the models presented in 2.5. PHY performance depends on the amount of data to be transmitted. Therefore, for UL, two different pack sizes are contemplated: 466 Bytes for 640x480 video and 520 Bytes for 1280x720 video. The TBS presented in Table 8 are calculated based on these sizes. As stated in section 3.2, the probability that a high-resolution video packet arrives is *a*. Thus, the *τ*_*tx*_ component in 42, which refers to the average transmission delay, is calculated by considering the packet size and its associated probability as follows:

$$\begin{array}{c} \tau_{tx}=a\cdot \frac{TBS_{1280x720}}{PHY_{Th}}+\left(1-a\right)\cdot \frac{TBS_{640x480}}{PHY_{Th}} \end{array}$$ (45)

The evaluation results for the UL direction are

$$Th_{PHY\_UL}=271packets/s$$

$$Delay_{PHY\_UL}=2.0360e-4s$$

On the other hand, DL transmission considers a single control command packet size. Therefore, its transmission delay is *τ*_*tx*_ = *TBS*_*CC*_/*PHY*_*Th*_. The evaluation performance results are

$$Th_{PHY\_DL_{A}}=50packets/s$$

$$Delay_{PHY\_DL_{A}}=1.900e-4s$$

and

$$Th_{PHY\_DL_{B}}=100packets/s$$

$$Delay_{PHY\_DL_{B}}=1.900e-4s$$

### 4.7 Overall 5G NR performance for remote driving

Tables 10–12 summarize the network performance results for the three transmission flows of 5G NR, which are relevant for the Remote Driving scenario. The overall cross-layer delay performance can be calculated by using Eq 1 as:

$$radio_{OWD}=4.8727e-04s\;\text{for}\;\text{UL}\;\text{Video}\;\text{Transmission}$$

$$radio_{OWD}=2.5181e-04s\;\text{for}\;DL_{A}$$

$$radio_{OWD}=2.5185e-04s\;\text{for}\;DL_{B}$$

**Table 10:** Performance evaluation results for UL video transmission.

	UL Video Transmission	
Protocol	Th (packets/s)	Delay (s)
SDAP	271 (Eq 3)	2e-8 (Eq 4)
PDCP	271 (Eq 29)	3.3582e-5 (Eq 19)
RLC	271 (Eq 31)	4.0309e-08 (Eq 32)
MAC	271 (Eq 33)	2.5002e-4 (Eq 34)
PHY	271 (Eq 41)	2.0360e-4 (Eq 42)
Cross-Layer	271 (Eq 2)	4.8727e-04 (Eq 1)

**Table 11:** Performance evaluation results for *DL*_*A*_ control packets transmission.

	*DL*_*A*_ *ControlPacketTransmission*	
Protocol	Th (packets/s)	Delay (s)
SDAP	50 (Eq 3)	2.0000e-08 (Eq 6)
PDCP	50 (Eq 29)	3.2945e-05 (Eq 19)
RLC	50 (Eq 31)	1.0000e-08 (Eq 32)
MAC	50 (Eq 33)	2.8762e-05 (Eq 34)
PHY	50 (Eq 41)	1.900e-04 (Eq 42)
Cross-Layer	50 (Eq 2)	2.5181e-04 (Eq 1)

**Table 12:** Performance evaluation results for *DL*_*B*_ control packets transmission.

	*DL*_*A*_ *ControlPacketTransmission*	
Protocol	Th (packets/s)	Delay (s)
SDAP	100 (Eq 3)	2.0000e-08 (Eq 6)
PDCP	100 (Eq 29)	3.2945e-05 (Eq 19)
RLC	100 (Eq 31)	1.0000e-08 (Eq 32)
MAC	100 (Eq 33)	2.8762e-05 (Eq 34)
PHY	100 (Eq 41)	1.900e-04 (Eq 42)
Cross-Layer	100 (Eq 2)	2.5185e-04 (Eq 1)

Considering that the performance requirements defined by the 5GAA [5] for Remote Driving establishes a maximum latency of 100 ms on UL and 20 ms on DL, it can be concluded that transmission over 5G NR introduces a very small latency over the data flow. Thus, the control station can be located further away from the gNB, as long as the latency is kept below the margins mentioned before. Therefore, our model can be used a starting point when analyzing if network architectures where the data flows across 5G NR, 5G transport network (TN), 5G core network (CN), and the Internet are feasible to deploy (e.g. see Fig 2a).

## 5 Simulation results

This section validates the results of the analytical model presented above. For this validation, a comparison with simulation results was used. The simulation uses the Julia language to write code that replicates the behavior of 5G NR protocols. Using this code, a series of iterations is run to obtain the average values of the performance metrics, throughput and latency. The parameters used for model evaluation and simulation are chosen to represent the transmission of control packets in the DL sense. This is chosen because it is the communication with the most stringent delay requirements and, therefore, must operate at conditions close to URLLC. A summary of the parameters used is presented in the Table 13.

**Table 13:** Simulation configuration parameters.

Parameter	Value
Traffic	1 ⋯ 200*packets*/*s*
SDAP Processing Delay	0.02*μs*
PDCP Integrity Delay	8.7660*μs*
PDCP Ciphering Delay	4.2107*μs*
PDCP Header Delay	0.02*μs*
RLC Haader Delay	0.02*μs*
RLC HARQ Transmission Delay	0.01*μs*
MAC Processing Delay	0.02*μs*
PHY Radio Distance	866*m*
PHY Bitrate	218*Mbps*

**Fig 11:**
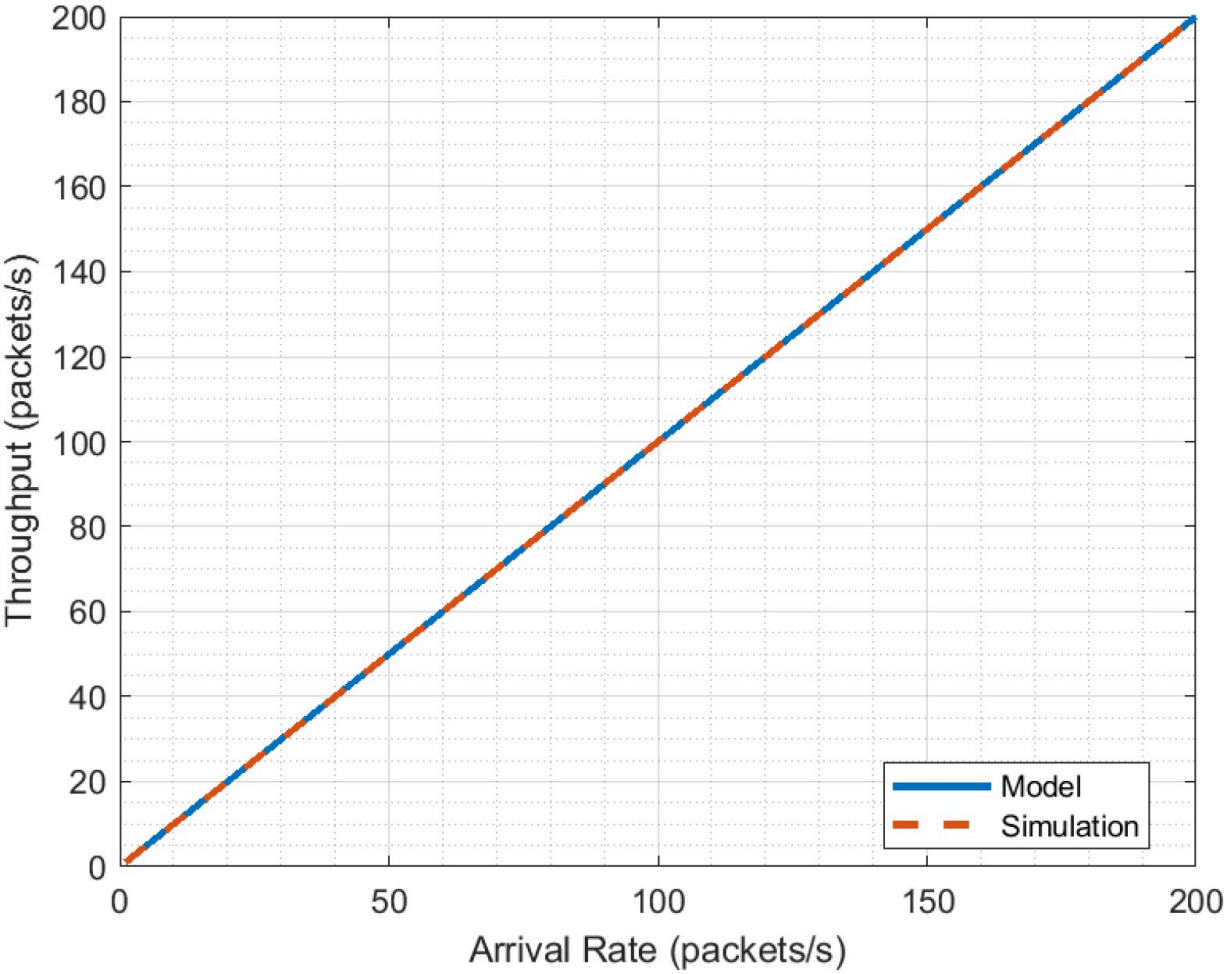
5G NR throughput evaluation.

**Fig 12:**
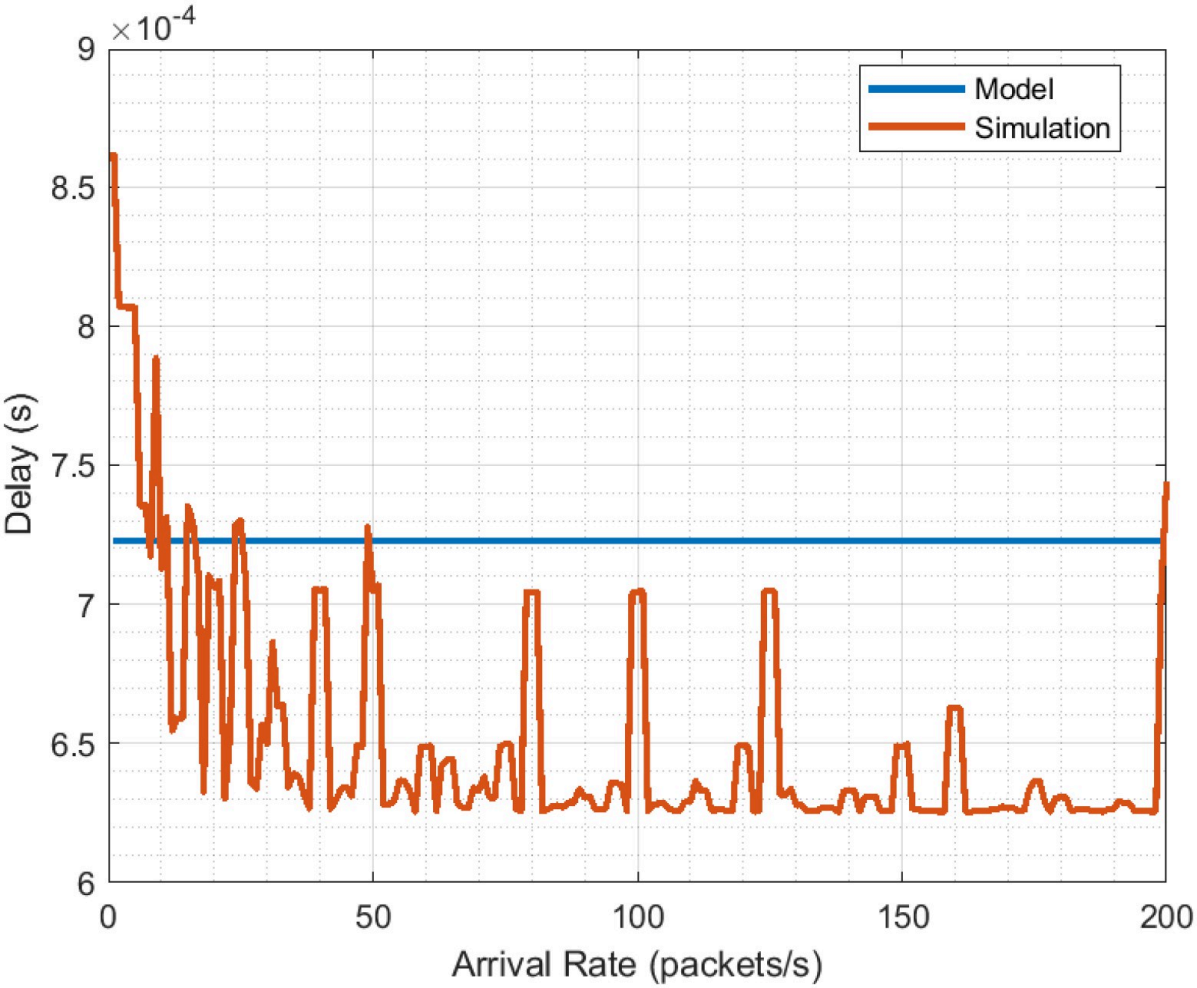
5G NR delay evaluation.

The processing delay values for the various layers are chosen to represent a device with sufficient capabilities to support 5G URLLC transmissions. The value of the radius, i.e., the distance between the vehicle and the gNB base station, is defined as in article [14], which presents a remote driving application. For the definition of the bit rate in the PHY layer, the maximum rate defined in [28] Section 5 is used. The analyzed traffic rate is chosen to cover the values of interest for the remote driving application, 50 and 100 packets/s. Using the values of the parameters in the Table 13, the performance results presented in Figs 11 and 12 are obtained for flow rate and latency, respectively.

**Fig 13:**
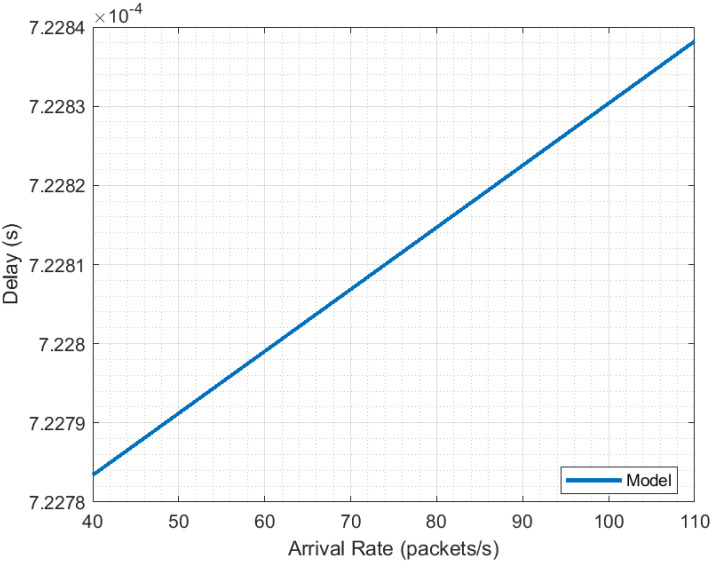
5G NR delay evaluation close view.

The presented performance evaluation results indicate that the model results adequately describe the behavior of 5G NR. This is based on the comparison of these with simulation results. The results for Flow (Fig 11) are practically identical, having a correlation of 0.9999 and an RMSE (*Root Mean Square Error* of 2.2919*e* − 4*packets*/*s*. On the other hand, although the simulation delay results seem more dispersed (correlation of −0.5155), both of them (model and simulation) share the same trend and their valuers are close, with an RMSE of 8.3321*e*−5*s*. This allows us to establish that the model is suitable for the performance evaluation of 5G NR. Although the delay results seem to have a constant value, a closer look reveals that it suffers from a slight increase, see Fig 13.

This finding suggests that not being careful in the rate of packets being transmitted may lead to the risk that delay grows beyond the thresholds required by URLLC applications. In addition, operating in non-ideal network conditions or where the scheduling mechanism is inadequate threatens compliance with URLLC delay requirements.

## 6 Discussion & conclusions

In this paper, we introduced an analytical model for the protocol stack of 5G NR. The model was designed using a layered approach that considers all protocols involved in 5G NR communication: SDAP, PDCP, RLC, MAC, and PHY. All the protocol functions concerning user data transmission were analyzed to develop the model. Thus, by using different analytical tools like queuing theory and SRN, the behavior of each radio protocol was described and its performance components were derived. These components can then be used to evaluate the performance provided by5G NR for different applications by calculating metrics like as delay and throughput with the model.

As an example of its utility, the derived model was used to evaluate the performance of an application with stringent delay requirements: Remote Driving. It was found that when the control station is located nearby the 5G gNB, the protocol stack of 5G NR does not introduces significant delay. Particularly, it was found that the accumulated delay for UL video transmission and control packets transmission is well below the maximum latency of 100 ms on UL and 20 ms on DL required by the 5GAA [5] for Remote Driving. Furthermoe, from this results it can be concluded that data transmission over 5g NR provides sufficient delay margin to locate the control station further away from the 5G gNB (e.g. see Fig 2a) and still meet the requirements set by the 5GAA for Remote Driving. As such, The model introduced in this paper model can complement end-to-end 5G analysis, such as the one presented in [14].

In conclusion, the model proposed in this paper can be used to evaluate the performance of the protocol stack of 5G NR under different network configurations, traffic conditions and scheduling mechanisms. By using the model, selecting an adequate parameter combination for different network conditions and applications is possible. Furthermore, the model can also be used to identify whether an edge, fog or cloud deployment is adequate for a given application. Thus, the developed model can be used as a starting point for end-to-end (E2E) network performance evaluations of 5G-supported deployments.
